# Comprehensive Profiling of Human Urinary Mercapturic Acid Conjugates Associated with Exposure to Reactive Chemical Species Using Enzymatic Deacetylation and High-Resolution Mass Spectrometry

**DOI:** 10.1021/acs.analchem.5c08181

**Published:** 2026-06-17

**Authors:** Yuan-Chih Chen, Man-Ni Zhuang, Jen-Yi Hsu, Yi-Chun Lin, Hsin-Yi Wu, Pao-Chi Liao

**Affiliations:** † Department of Environmental and Occupational Health, College of Medicine, 38026 National Cheng Kung University, Tainan 701401, Taiwan; ‡ Instrumentation Center, 33561 National Taiwan University, Taipei 10617, Taiwan

## Abstract

Glutathione conjugation is a key metabolic detoxification pathway, and its downstream products, mercapturic acid conjugates (MACs), may serve as urinary biomarkers of toxicant exposure. However, MAC profiling is hindered by incomplete filtering coverage and the lack of comprehensive spectral and structural databases. To address these limitations, we developed an analytical workflow integrating novel enzymatic deacetylation with ultrahigh-performance liquid chromatography-high-resolution mass spectrometry (UHPLC-HRMS)-based neutral loss filtering. The aminoacylase-1-mediated deacetylation provides an orthogonal approach to detect MACs that lack the characteristic neutral loss of 129.0426 Da. Additionally, an in-house MAC library of 734,170 putative structures, combined with an *in silico* structure annotation tool, facilitated efficient MAC identification. The strategy was validated using 11 MAC standards spiked into urine and applied to urine samples from 15 participants before and after consuming deep-fried foods. A total of 847 features were mapped to at least one MAC structure, exceeding the ∼100 reported in previous studies, with 581 absent from the Human Metabolome Database (HMDB) or PubChem. Postconsumption, 59 MACs increased and 11 decreased, reflecting exposure-related shifts in reactive aldehydes, lipid oxidation products, acylcarnitines, and acyl-CoA intermediates. These findings demonstrate the utility of the workflow for capturing urinary MAC changes associated with deep-fried food consumption and for prioritizing candidate exposure indicators of reactive chemical species for future validation studies.

## Graphical abstract

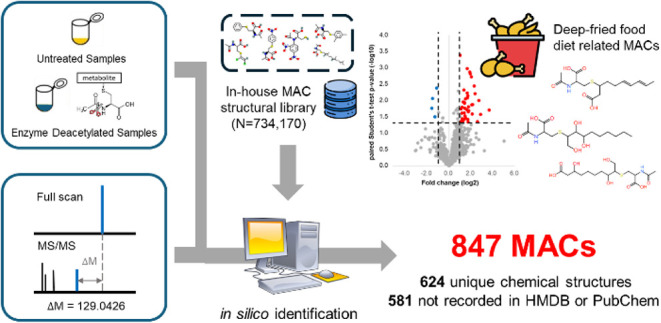

## Introduction

Humans are estimated to be exposed to one to three million chemical compounds throughout their lifetimes.^1–3^ Among these environmental exposures, reactive chemical species,^4,5^ or electrophilic compounds, pose an even greater risk due to their tendency to bind with nucleophilic macromolecules, such as DNA, RNA, and proteins, forming adducts that can disrupt biological processes,^6–8^ which subsequently lead to the development of cancer and chronic degenerative diseases.^9,10^ To counteract the deleterious effects of electrophilic xenobiotics, the body employs glutathione (GSH) conjugation.^11–13^ This conjugation prevents electrophilic species from covalently binding to DNA or proteins, thereby mitigating their toxic effects.^14^ Glutathione (GSH) forms a thioether bond with an electrophile, catalyzed by glutathione S-transferases (GSTs).^15^ These glutathione conjugates are further converted into mercapturic acid conjugates (MACs)^15^ and excreted in urine, making them a feasible target for biomonitoring to evaluate exposure to electrophilic xenobiotics.^16^ Previous studies have utilized MACs as biomarkers of exposure to reactive chemicals. For example, *N*-acetyl-S-phenyl-l-cysteine (SPMA) is commonly used as a urinary biomarker to assess benzene from ubiquitous industrial and environmental sources of exposure.^17,18^

Most of the published studies focused on biomonitoring for the MACs of specific target toxic substances, utilizing neutral loss scan mode in triple quadrupole mass spectrometers (QqQ).^19,20^ Recently, the development of high-resolution mass spectrometry (HRMS)^21,22^ and nontargeted analytical approaches^23^ allows the identification of unknown chemicals without the need for chemical standards.^24^ Combined with data-independent acquisition (DIA)^25,26^ and neutral loss filtering techniques,^27^ the HRMS-DIA-based nontargeted approach has become a powerful tool for discovering conjugated metabolites in biological samples.^28^ Comprehensive biomonitoring of MACs, or the MAC “adductome”, can facilitate the identification of previously unrecognized reactive chemical species while expanding coverage for monitoring general exposure to reactive chemical species.^29^

Jamin et al. developed a nontargeted approach using DIA analysis for monitoring the specific neutral loss of mercapturates.^30^ By filtering out signals with the characteristic neutral loss of 129.0426 Da (C_5_H_7_NO_3_), they were able to detect 21 MACs from oxidized polyunsaturated fatty acids in urine. Another study by Xie et al. demonstrated an expanded profiling of urinary MACs, successfully annotating 116 urinary MACs, including 25 previously unreported structures.^31^ Chen et al. applied a similar strategy and identified 146 MACs in human urine at confidence level 3 or higher.^32^ Although instrumentation limitations have been overcome, certain challenges in nontargeted urinary MAC profiling have not yet been solved. For instance, some MACs do not exhibit the aforementioned characteristic neutral loss.^31,33,34^ Although many MACs exhibit the characteristic neutral loss of 129.0426 Da, this behavior is not universal. While the available literature has not yet established a comprehensive family-level classification of MACs lacking this neutral loss, our validation data suggest that dithiocarbamate-related MACs may represent one such subgroup. Consequently, relying strictly on neutral loss filtering inherently restricts the observable MAC exposome and necessitates the use of orthogonal screening techniques. In addition, there are only a small number of MACs listed in existing databases, limiting the coverage of identification.^31^

In this study, an analytical strategy incorporating enzymatic deacetylation with an HRMS-DIA-based neutral loss filtering approach was developed to expand the coverage of human urinary MAC profiling associated with exposure to reactive chemical species. The novel enzymatic deacetylation strategy provides an orthogonal approach for filtering MACs that lack the characteristic neutral loss. An in-house MAC structural library with over 700,000 putative MACs was also constructed to broaden the scope of MAC structure annotation. As proof of concept, the proposed workflow was applied to profile MACs in participants’ urine before and after consuming deep-fried foods, a known source of exposure to electrophilic compounds.^35,36^

## Experimental Section

### Chemicals and Materials

Eleven MAC standards (purity ≥ 99%) for method development were purchased from Toronto Research Chemicals (Canada) and Cayman Chemical (Ann Arbor, MI, USA). Five MAC standards (purity ≥ 99%) for structural identification were purchased from Toronto Research Chemicals (Canada). Sodium phosphate dibasic, sodium phosphate monobasic (purity ≥ 99%), and acetonitrile (ACN, LC-MS grade) were purchased from J.T. Baker (Phillipsburg, NJ, USA). Aminoacylase-1 from porcine kidney (acylase I, ≥1500 units/mg), formic acid (LC-MS grade), methanol (MeOH, LC-MS grade), sodium hydroxide (NaOH, LC-MS grade), hydrochloric acid (HCl, LC-MS grade), and four nonconjugated internal standards (purity ≥ 99%), including chloramphenicol, sulfadimethoxine, ketoprofen, and diclofenac sodium salt, were purchased from Sigma-Aldrich (St. Louis, MO, USA).

### Sample Collection

Over the course of 2 weeks, the midstream first-morning urine samples were collected from 15 participants (11 males and 4 females, recruited from the authors’ research institute) of similar age, using brown, opaque 250 mL polypropylene bottles. Demographic information, including age, gender, height, weight, and BMI, was obtained through questionnaires and is detailed in Supporting Information Table S1. Participants were instructed to avoid consuming deep-fried foods during the first week. In the subsequent week, deep-fried foods were distributed to each participant from the first day to the fourth day. During these 4 days, participants were provided with approximately 200–300 g of deep-fried foods daily, including fried chicken, fried king oyster mushrooms, and fried sweet potatoes. Detailed frying conditions were not recorded in a standardized manner for source-specific exposure reconstruction in this proof-of-concept study. The urine samples were collected on the fourth and fifth days of both weeks. Informed consent was obtained from all participants, and the study protocol was approved by the Institutional Review Board of the National Cheng Kung University Hospital, Tainan, Taiwan (IRB number: AER-110-489).

### Enzymatic Deacetylation

The urine samples were adjusted to pH 7.4 using 1 N NaOH or 1 N HCl and centrifuged at 20,000 × g at 4 °C for 30 min. The resulting supernatants were diluted 5-fold with 100 mM sodium phosphate buffer (pH 7.4), prepared from sodium phosphate dibasic and sodium phosphate monobasic. Aminoacylase-1 powder was dissolved in 100 mM sodium phosphate buffer to a unit of 5 U/μL. For each urine sample, two incubation tubes were prepared: Tube 1 contained 220 μL of 5-fold diluted urine, 132 μL of sodium phosphate buffer, and 48 μL of aminoacylase-1, resulting in a total incubation volume of 400 μL with a final enzyme amount of 240 U, while Tube 2 replaced 48 μL of aminoacylase-1 with 48 μL of sodium phosphate buffer, maintaining the same total incubation volume of 400 μL. For the ten replicate experiments, 132 μL of sodium phosphate buffer was replaced with an 11-MAC standard mixture, ensuring a final concentration of 10 μM for each MAC in the 400 μL incubation volume. The mixtures were incubated at 37 °C for 24 h, and the reactions were terminated by transferring 100 μL of the incubation mixture into 100 μL of cold ACN (−20 °C). The reaction mixtures were centrifuged at 20,000 × g at 4 °C for 30 min. Subsequently, 10 μL of a nonconjugated internal standard mixture was added to 90 μL of the supernatants, achieving a final concentration of 200 ppb for each of the four nonconjugated internal standards. The samples were stored at −20 °C before subsequent analysis, and for quality control, a pooled urine sample comprising all urine samples was also prepared and periodically injected throughout the analytical sequence to monitor stability.

### Ultra-High-Performance Liquid Chromatography (UHPLC)-HRMS-DIA Analysis and Data Processing

UHPLC-HRMS analyses were conducted using a Dionex UltiMate 3000 UHPLC system coupled with an Orbitrap Q Exactive Plus (Thermo Fisher Scientific, Bremen, Germany). A Luna Omega Polar C18 column (100 × 2.1 mm, 1.6 μm, Phenomenex, Torrance, CA, USA) was used and maintained at 40 °C. After loading 5 μL of the sample into the column, analyte separation was performed at a flow rate of 300 μL/min with the following eluent gradient: 2% B for 0–1 min; 2–95% B in 1–12 min; 95% B in 12–14 min; 95–2% B in 14–15 min; 2% B for 15–16 min. Solvent B was 100% ACN containing 0.1% formic acid, while solvent A was 2% ACN in deionized water containing 0.1% formic acid. For DIA analysis, full scan events were performed from 80 to 800 *m*/*z* in negative ion mode at a resolution of 70,000 full width at half maximum (fwhm), with an automatic gain control (AGC) target of 3 × 10^6^ and a maximum injection time of 200 ms. For MS/MS events, six isolation windows, each with a width of 120 *m*/*z*, were performed with normalized collision energy (NCE) of 30% and 60% in stepped mode. The AGC target for MS/MS was set at 1 × 10^6^, with a resolution of 17,500 fwhm.

Peak picking, peak alignment, and chromatographic deconvolution of raw UHPLC-HRMS-DIA data were performed using MS-DIAL software (ver. 4.9.2). The parameters were configured as follows: the tolerance for MS1 mass accuracy was 0.001 Da, the tolerance for MS2 mass accuracy was 0.005 Da, the minimum peak height was 10 000 amplitudes, and the mass slice width was 0.002 Da. The alignment reference file was derived from the QC sample, with a retention time (RT) tolerance of 0.2 min and an MS1 tolerance of 0.001 Da. To account for the variability among different urine samples, the raw abundance of each feature in the alignment table was first divided by the sum of abundance of all features to normalize the variations in urine concentration.^37^ The data were normalized again by using multiple internal standards to eliminate matrix effects among samples. Each MAC candidate was normalized by the nearest internal standard in RT using eq 1:^38^

1 $$A_{\mathrm{normalized}}=A_{\mathrm{raw}}\frac{IS_{\mathrm{average}}}{IS_{\mathrm{raw}}}$$

*A*_normalized_ and *A*_raw_ represent the normalized and raw peak abundances of each MAC candidate, respectively. IS_average_ and IS_raw_ refer to the average and raw peak abundances of the nearest internal standard in the RT.

## Results and Discussion

### Overall Study Design and Method Development

The study design for nontargeted urinary MAC profiling is illustrated in Figure 1 and consists of the following three stages: (i) method development of MAC profiling integrating enzymatic deacetylation with HRMS-DIA, (ii) method validation of 11 MAC standards spiked into urine samples, and (iii) application of this method to profile urinary MACs before and after consumption of deep-fried foods. The detailed results of each step in the study design are described in the following sections. To accurately account for complex matrix effects and interferences inherent to biological samples, spiked urine samples from a single volunteer were utilized as the starting point for the method development and validation stages (stages i and ii), ensuring that the workflow was optimized for real-life conditions before being applied to the deep-fried food exposure study (stage iii).

**Figure 1:**
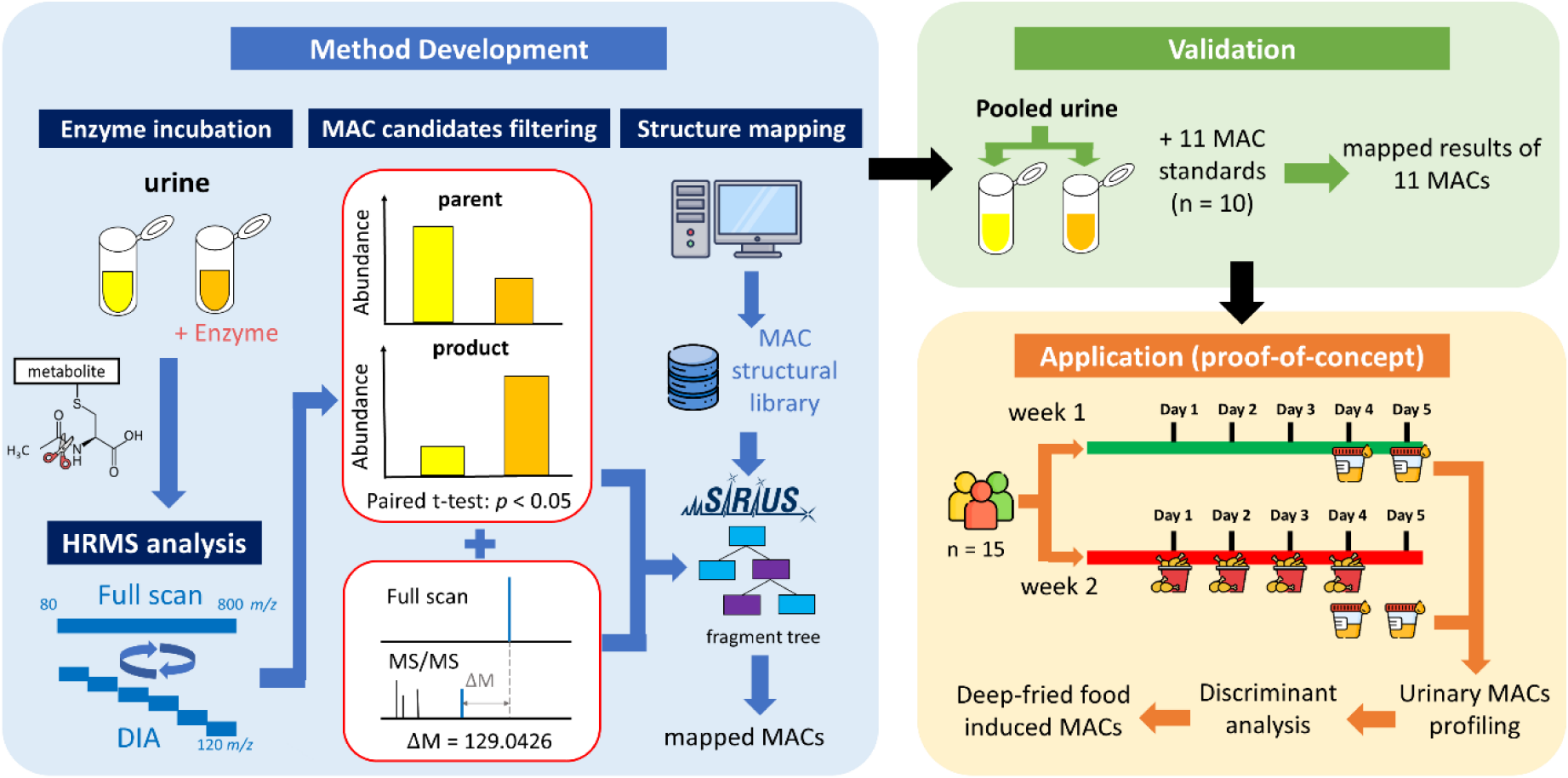
The study design for profiling and identification of MACs.

### Enzymatic Deacetylation

An effective strategy to enhance the coverage of MAC identification involves integrating enzymatic treatment with HRMS-based metabolomics approaches.^39^ Enzymatic deconjugation offers an orthogonal approach for refining the detection of conjugated metabolites by selectively removing conjugate moieties. In addition to expanding the pool of detectable conjugated metabolite candidates,^40^ enzymatic treatment can also help reduce false positives and improve the quality of DIA-derived MS/MS spectra for these metabolites.^28^ Although no commercially available enzyme currently achieves complete hydrolysis of all MAC species, several enzymes demonstrate substrate specificity toward functional groups commonly found in MACs. Among them, aminoacylase-1 (EC 3.5.1.14) is a zinc-dependent enzyme known to catalyze the hydrolysis of *N*-acyl-L-amino acids, producing free L-amino acids and their corresponding carboxylic acids (e.g., acetyl groups). According to UniProt (Q03154), aminoacylase-1 possesses broad substrate specificity and is capable of deacetylating various N-acetylated compounds, including MACs. This enzymatic activity has been validated by prior studies^41,42^ and is documented in the KEGG reaction database (reaction R10553), which describes the enzyme’s ability to catalyze the deacetylation of acetylated cysteine conjugates.

In the development stage, control urine samples were first prepared in both enzyme-treated and untreated forms to establish baseline enzymatic interactions within a biological matrix. Enzymatic hydrolysis of conjugated metabolites has been shown to expand the number of identified conjugated metabolites when combined with neutral loss filtering.^28,39^ However, no established or commercially available enzymatic solution currently exists for the deconjugation of MACs. This study developed an innovative strategy for the deacetylation of MACs using aminoacylase-1. While aminoacylase-1 is limited to removing the acetyl group from MACs rather than fully cleaving the mercapturic acid conjugate, it provides a complementary approach for MAC screening. By screening MAC candidate features that significantly decrease and deacetylation products that significantly increase after enzymatic deacetylation, this method offers an orthogonal strategy to enhance MAC screening beyond relying solely on neutral loss filtering.

Two critical parameters—enzyme unit and incubation time—were systematically investigated to determine the workable conditions of enzymatic deacetylation. For the enzyme unit, spiked urine samples were treated with varying amounts of aminoacylase-1 (80 U, 160 U, 240 U, 320 U, and 400 U per sample), with three replicates for each unit. The results showed that the intensities of 11 MAC standards decreased markedly at enzyme concentrations above 80 U, indicating effective deacetylation (Figure S1). At 240 U, the depletion of some MACs began to plateau. Based on these findings, 240 U of aminoacylase-1 was selected as the enzyme unit for all subsequent experiments, ensuring efficient and complete deacetylation without excess enzyme. The effect of incubation time on the enzymatic deacetylation was studied through a time-course experiment, focusing on the deacetylation of 11 MAC standards. The results showed that 9 out of 11 MAC standards exhibited significant decreases in abundance at 24 h of incubation, with the extent of deacetylation increasing over time. By 48 h, the remaining two MACs—MPhMA and 5HPnFuMA—also showed significant reductions. Although a 48-h incubation appears to enhance overall reaction efficiency, previous studies^43^ have reported that prolonged storage at or above room temperature may lead to MAC degradation. Moreover, a 48-h incubation period presents practical challenges for experimental scheduling and workflow efficiency. Therefore, the incubation time for all subsequent experiments was standardized at 24 h to ensure reproducibility and maximize the efficiency of the deacetylation process.

### HRMS-DIA Analysis and MAC Candidates Filtering

For HRMS-DIA analysis, a neutral loss filtering of 129.0426 Da was employed, with a mass tolerance set at 5 ppm. To expand the range of collision-induced dissociation (CID), two different NCEs, 30% and 60%, were applied in a stepped mode^28^ to combine fragments from medium and high NCEs into a single MS/MS spectrum. DIA analyses were performed with varying isolation windows of 60, 80, and 120 *m*/*z*, along with an all-ion fragmentation (AIF) analysis, and the results were compared. Among these, the DIA analysis with an isolation window of 120 *m*/*z* generated a total of 20,664 features, making it the second most productive in terms of feature identification, just behind the AIF analysis, which yielded 21,106 features (Figure S3). Despite the slightly higher feature count of the AIF analysis, the DIA analysis provided higher-quality MS/MS spectra with significantly reduced noise (Figure S4). Given these advantages, DIA analysis with the 120 *m*/*z* isolation window was chosen as the preferred scan mode, offering a balance between feature identification and data quality.

It should be noted that the use of a relatively wide 120 *m*/*z* DIA isolation window may increase the probability of coselecting coeluting ions. Such coisolation can generate more complex MS/MS spectra and may lead to fragment-ion misassignment, particularly in chemically complex urinary samples. To minimize this effect, chromatographic deconvolution was performed using MS-DIAL, which assigns fragment ions to precursor features based on their chromatographic coelution profiles. In addition, neutral loss filtering was conducted using a strict 5 ppm mass tolerance, and candidate spectra were further evaluated by SIRIUS-based structural annotation to improve the reliability of the feature assignment. Nevertheless, coisolation cannot be completely eliminated in DIA-based acquisition, especially when a wider isolation window is used. Therefore, although the 120 *m*/*z* DIA method increased coverage while maintaining an acceptable spectral quality, the resulting annotations were conservatively reported as tentative candidates unless confirmed by authentic standards.

For MAC candidates filtering, features of potential MAC candidates were filtered based on at least one of the following criteria: (1) significant changes in feature abundances between enzymatically treated and untreated samples (defined as a paired Student’s *t* test *p*-value <0.05, with a fold change <0.5 for parent compounds or >2 for deacetylated products) or (2) the presence of a characteristic neutral loss of 129.0426 Da specific to MACs. For filtering based on feature abundance changes, two conditions were considered: 1. Parent compound filtering (upper part in the “MAC candidates filtering (1)” section of Figure 1): Parent compounds of MACs were expected to undergo deacetylation by the enzyme, leading to a decrease in abundance after enzymatic deacetylation. The filtering criterion required a statistically significant decrease in normalized feature abundance (paired Student’s *t* test, *p*-value <0.05, and fold change <0.5) between treated and untreated samples. 2. Deacetylated product filtering (lower part in the “MAC candidates filtering (1)” section of Figure 1): Deacetylated products of MACs, generated by enzymatic activity, were expected to exhibit an increase in abundance after enzymatic deacetylation. The filtering criterion required a statistically significant increase in normalized feature abundance (paired Student’s *t* test, *p*-value <0.05, and fold change >2) between treated and untreated samples. Once a deacetylated product candidate (*m*/*z* = C_d_) was found, the paired parent MAC candidate was determined by both matching the *m*/*z* value of the paired parent MAC candidate (*m*/*z* = C_d_ + 42.0106) and confirming that the normalized feature abundance of the paired parent MAC candidate did not significantly increase after enzymatic deacetylation. Two types of blanks—solvent blank and enzyme reaction blank—were included to eliminate false positive MAC candidates showing no significant abundance difference between urine and blank samples.

For filtering based on characteristic neutral loss, each precursor ion (*M*_*c*_) in UHPLC-HRMS data was evaluated by comparing its *m*/*z* value to that of its corresponding fragment ion (*M*_*cf*_). A precursor was considered a potential MAC candidate when it satisfied eq 2,

2 $$\left|M_{c}-M_{cf}-k\right|\leq z$$

In this equation, *k* represents the characteristic neutral loss mass of MACs (129.0426 Da) and *z* denotes the tolerance for measurement uncertainty, which was set at 5 ppm. MAC candidate features filtered through enzymatic deacetylation and neutral loss filtering were subsequently combined into a union set for structural annotation.

### MAC Structural Database Building and Structural Annotation with SIRIUS

An in-house MAC structural database was developed to facilitate *in silico* structural annotation of both known and unknown MACs. The library construction process, illustrated in Figure 2, commenced with the compilation of compound structural information from three online databases: HMDB,^44^ Tox21,^45^ and T3DB.^46^ These databases encompass small molecule compounds naturally present in the human body, as well as toxic substances, including reactive chemical species that humans may be exposed to. The structures were downloaded in the SMILES format and subsequently screened to exclude compounds that already contained glutathione or mercapturic acid moieties. The remaining unconjugated structures were then processed using two biotransformation prediction tools, BioTransformer^47^ (version 3.0.0) and GLORYx^48^ (version 1.0.8), to predict the possible glutathione conjugation products. The biotransformation parameters were set to allow 0–3 steps for phase I reactions and 1 step for glutathione conjugation. The predicted glutathione conjugates in the SMILES format were further processed using an in-house program equipped with customized SMILES arbitrary target specification (SMARTS) to convert glutathione conjugates into their corresponding MACs. All of the generated MAC structures were validated for structural consistency and proper connections of the mercapturic acid groups using the RDKit toolkit^49^ in Python, including molecular parsing (function MolFromSmiles), valence verification, and full molecule sanitization (function SanitizeMol with SANITIZE_PROPERTIES). For compounds that already contained MACs, their SMILES representations were directly incorporated into the MAC structural database. Compounds conjugated with glutathione were converted into their corresponding MACs by the in-house program before being integrated into the library. In addition, a reported MAC structural database, comprising 220 previously reported MAC structures,^31^ was also integrated into the MAC structural database built in this study.

**Figure 2:**
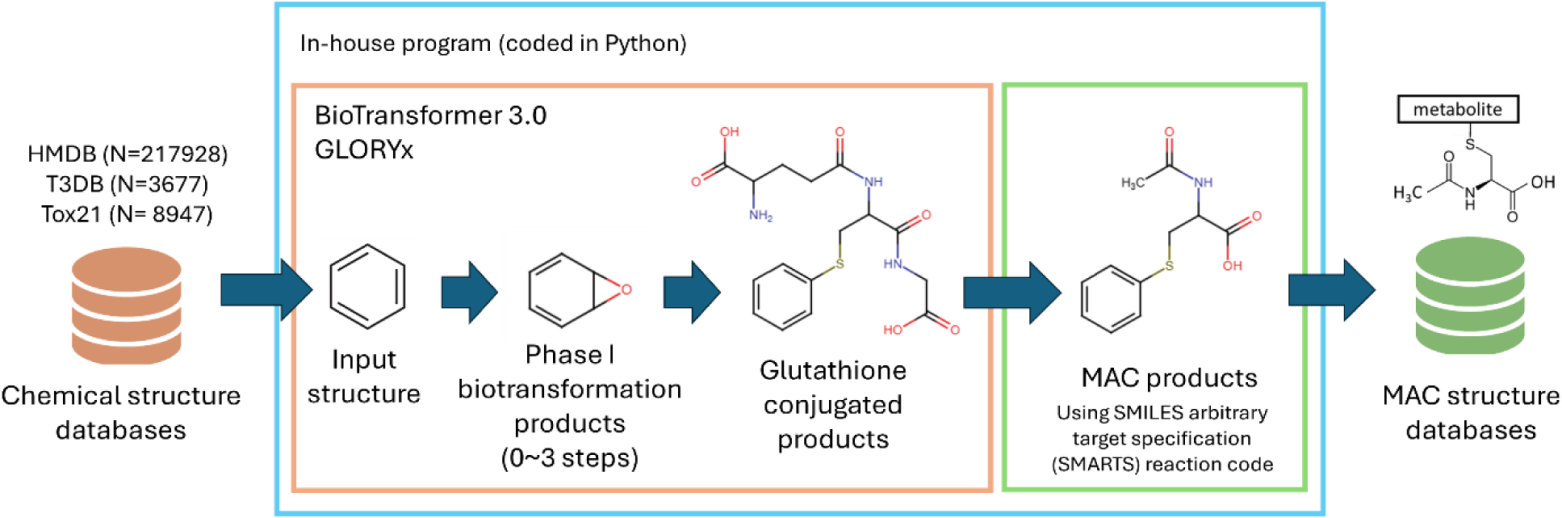
The process of building the in-house MAC structural library. Chemical structure data from three databases were processed using two biotransformation prediction tools to generate potential glutathione conjugation products. These products were subsequently transformed into MAC structures and integrated into the in-house MAC structural database.

In total, 734 170 MAC structures were generated and compiled into the MAC structural database, each represented by a unique standardized SMILES string to ensure consistent and unambiguous molecular identification. Among these, 62 689 MACs originated from multiple parent compounds due to the application of generalized phase I and II biotransformation rules. These redundancies were explicitly annotated to maintain traceability. When such MACs are annotated in experimental data, all plausible parent compounds are reported to reflect inherent structural ambiguity. The generation of a large number of MAC candidate structures in the database aims to maximize chemically plausible MAC coverage and improve the detection of unknown species, aligning with recent large-scale metabolomics and exposomics approaches.^50,51^

The structure annotation of urinary MACs was performed using SIRIUS CSI:FingerID (version 6.0.7).^52,53^ The deconvoluted DIA-MS/MS spectra of filtered MAC candidates from each untreated urine sample were imported into the software in the “.mat” format. Molecular formulas and fragmentation trees for all candidates were generated based on the [M – H]^−^ adduct, with a mass tolerance of 5 ppm, including the following elements: C, H, O, N, P, S, F, Cl, and Br. Isotope patterns were considered during molecular formula calculation. The assigned molecular formulas and fragmentation trees were subsequently compared with molecular fingerprints derived from structural databases, including the in-house MAC structural database, HMDB, and PubChem. HMDB and PubChem served as “baseline databases” to minimize the misidentification of non-MAC structures as MACs. The annotation results were exported, presenting the top structural candidate^54,55^ for each feature and reporting both the structure of the identified MAC and the name of the parent compound.

### Validating the Developed Strategy Using 11 MAC Standards

The feasibility of the developed approach was validated with 11 commercially available MAC standards (Table 1) spiked into ten replicates of urine samples from one volunteer. The structures and SMILES codes of the MAC standards are listed in Supporting Information Table S2. The two characteristics of the 11 MACs, a neutral loss of 129.0426 Da and a significant abundance change after enzymatic deacetylation, were systematically investigated. The precursors of all 11 MACs were detectable by UHPLC-HRMS in untreated urine samples, with 7 of them significantly decreased in abundance (Figure 3a) (paired Student’s *t* test, *p-*value <0.05, and fold change <0.5), while 9 exhibited the characteristic neutral loss, 129.0426 Da. The two MACs that did not exhibit the characteristic neutral loss displayed the characteristic changes in signal abundance. Therefore, the union of the two characteristics was able to cover all 11 MAC standards (Figure 3b).

**Table 1:** List of 11 MAC Standards with Their Mass Spectrometry Information

Name of MAC standards	Abbreviation	Molecular formula	Theoretical *m*/*z*	Characteristic neutral loss	Characteristic changes in signal abundance^a^
*N*-Acetyl-S-phenyl-l-cysteine	SPMA	C_11_H_13_NO_3_S	238.0543	X	X, Δ
*N*-Acetyl-S-benzyl-l-cysteine	BzMA	C_12_H_15_NO_3_S	252.0700	X	Δ
*N*-Acetyl-S-(1,2-dichloroethenyl)-l-cysteine	12CEMA	C_7_H_9_Cl_2_NO_3_S	255.9607	X	X
*N*-Acetyl-S-(2,4-dimethylphenyl)-l-cysteine	MPhMA	C_13_H_17_NO_3_S	266.0856	X	
*N*-Acetyl-S-(4-nitrophenyl)-l-cysteine	4NPhMA	C_11_H_12_N_2_O_5_S	283.0394	X	X, Δ
*N*-Acetyl-S-(trichlorovinyl)-l-cysteine	122CVMA	C_7_H_8_Cl_3_NO_3_S	289.9218	X	X, Δ
*N*-Acetyl-S-(tetrahydro-5-hydroxy-2-pentyl-3-furanyl)-l-cysteine	5HPnFuMA	C_14_H_25_NO_5_S	318.1381	X	Δ
*N*-Acetyl-S-[*N*-(2-phenylethyl)thiocarbamoyl]-l-cysteine	2PhECaMA	C_14_H_18_N_2_O_3_S_2_	325.0686		X
*N*-Acetyl-S-(2,4-dinitrophenyl)-l-cysteine	24NPhMA	C_11_H_11_N_3_O_7_S	328.0245	X	
*N*-Acetyl-S-[[[4-(methylsulfinyl)butyl]amino]thioxomethyl]-l-cysteine	4MSfBAToMMA	C_11_H_20_N_2_O_4_S_3_	339.0512		X
*N*-Acetyl-S-trans,trans-farnesyl-l-cysteine	AFC	C_20_H_33_NO_3_S	366.2108	X	X, Δ

a X: paired Student’s *t* test, *p*-value <0.05, and fold change <0.5. Δ: matched with a deacetylation product satisfying *m*/*z*(parent) = *m*/*z*(product) + 42.0106, while the normalized feature abundance of the parent feature did not significantly increase after treatment.

**Figure 3:**
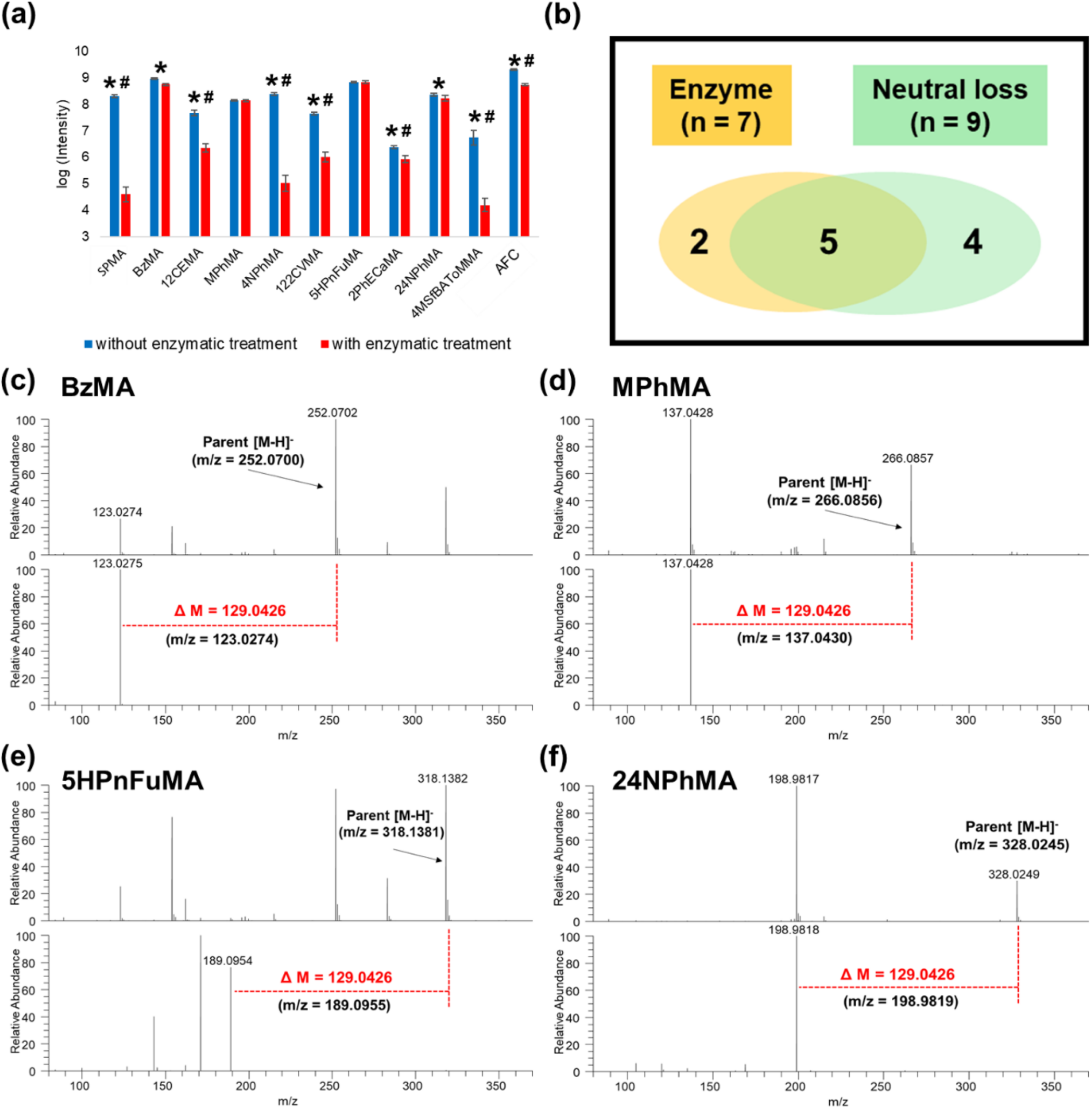
Validation of the developed approach using 11 MAC standards. (a) Significant abundance decrease (*: *p*-value <0.05, #: fold change <0.5) was observed for 7 MACs after enzymatic treatment. (b) Integration of two criteria—enzyme treatment and neutral loss—successfully annotated all 11 MAC standards. (c, d, e, f) Neutral loss of 129.0426 Da was confirmed for BzMA, MPhMA, 5HPnFuMA, and 24NPhMA, respectively.

For the enzymatic deacetylation, 7 of the 11 standards exhibited a statistically significant decrease in signal abundance following enzymatic deacetylation. Five of the 11 standards appeared simultaneously with the characteristic neutral loss of mercapturic acids. Among the other four MAC standards—BzMA, MPhMA, 5HPnFuMA, and 24NPhMA—that did not exhibit the characteristic abundance changes, their MS/MS spectra displayed the characteristic neutral loss (Figure 3c–f). These results indicate that the aminoacylase-catalyzed deacetylation reaction occurred and that the enzymatic efficiency in removing acetyl groups varied across different MACs. Furthermore, the deacetylation products of 6 out of the 11 MAC standards, including BzMA and 5HPnFuMA, showed a statistically significant increase in signal abundance (Supporting Information Figure S1 and S2). These two standards showed significant increases in the signal abundance of deacetylation products even though their parent compounds did not exhibit a significant decrease. This finding suggests that detecting deacetylation products can serve as a complementary strategy to identify MACs that may not undergo obvious deacetylation, thereby expanding the scope of screening results.

For the neutral loss filtering, 2 out of 11 MACs, 2PhECaMA and 4MSfBAToMMA, did not exhibit the expected neutral loss. Further investigation into the relationship between NCE and the neutral loss behavior of these two MACs revealed that signals indicative of neutral loss were observed only at low NCEs of 10% and 20% (Figure 4b). The reason behind this rapid signal decay is likely that the dithiocarbamate structure in these two MACs renders the conjugate highly unstable, leading to extensive or alternative fragmentation pathways rather than the typical 129 Da neutral loss when exposed to moderate to high collision energies.^56^ This observation highlights that certain MACs may not consistently exhibit a characteristic neutral loss of 129.0426 Da in their MS/MS spectra under some conditions. Importantly, these two MACs, which could not be filtered through neutral loss filtering, were successfully captured using an enzymatic deacetylation strategy. This finding highlights the complementarity of these two orthogonal approaches, demonstrating their potential for the comprehensive profiling of urinary MACs.

**Figure 4:**
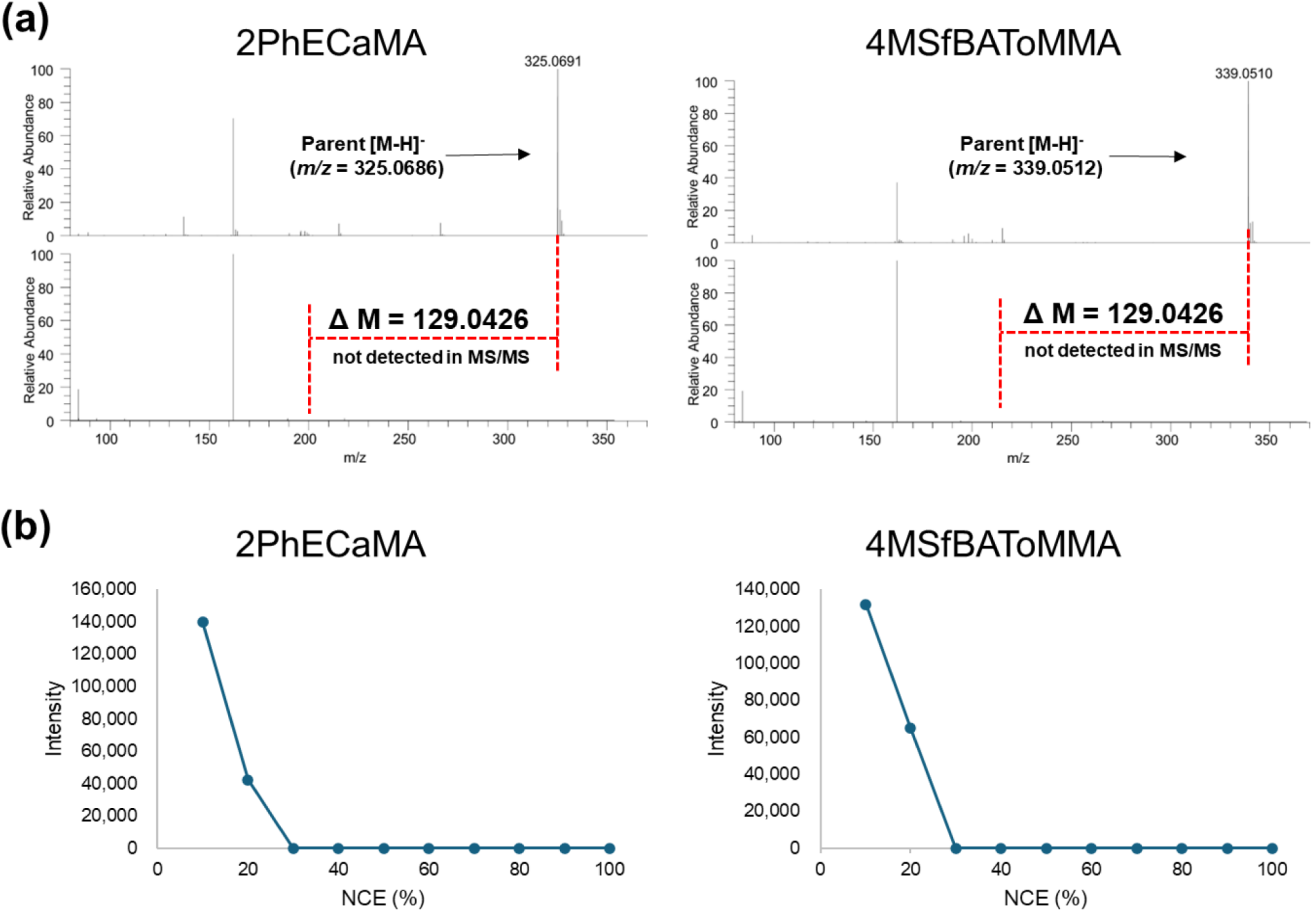
Relationship between NCE and the neutral loss behavior of 2PhECaMA and 4MSfBAToMMA. (a) The MS and MS/MS spectra at NCE = 30% of the two MAC standards showing the absence of fragments corresponding to the characteristic neutral loss of 129.0426 *m*/*z*. (b) The MS/MS abundance for the neutral loss of 129.0426 *m*/*z* at different NCEs.

The collision-energy-dependent behavior of 2PhECaMA and 4MSfBAToMMA further suggests that acquisition energy should be considered when designing nontargeted MAC profiling workflows. These two dithiocarbamate-related MACs exhibited the characteristic neutral loss of 129.0426 Da only at low collision energies (NCE 10–20%), whereas the signal disappeared at higher NCEs. This finding indicates that labile MAC subclasses may undergo extensive or alternative fragmentation under moderate-to-high collision energies, thereby reducing the sensitivity of neutral-loss-based screening. Future workflows may therefore benefit from incorporating an additional low-NCE DIA acquisition step, such as NCE 10–20%, to improve the detection of labile MACs. However, this strategy should be optimized carefully because additional DIA events would increase the duty cycle and may reduce the number of data points acquired across chromatographic peaks.

For structural annotation, 8 of the 11 MAC standards matched the top structural candidate presented by SIRIUS. While the remaining three MACs, MPhMA, 5HPnFuMA, and 4MSfBAToMMA, did not match their top candidate, the annotation results suggested similar isomers of true structures (Table S3). For MPhMA, the top candidate was a constitutional isomer of MPhMA. Two methyl groups in the meta-position were misidentified as an ethyl group on the benzene ring. For 5HPnFuMA, the top candidate corresponded to the product of 5HPnFuMA after its five-membered heterocyclic ring underwent ring opening. For 4MSfBAToMMA, the positions of the nitrogen and sulfur on the dithiocarbamate were swapped. Despite these discrepancies, the annotation process demonstrated its ability to identify MAC structures with a reasonable level of confidence.

### Application in Deep-Fried Foods Study

Deep-fried foods are widely consumed due to their appealing taste, affordability, and convenience in preparation. During the frying process, various reactive chemical species—including aldehydes, epoxy-fatty acids, ketones, hydrocarbons, carboxylic acids, furans, pyrazines, and pyridines—are generated through complex chemical reactions and can permeate into the deep-fried foods.^35,57^ Many of these ingested reactive chemical species are believed to be metabolized and excreted from the human body in the form of MACs. In this study, a comparative analysis of urine samples collected from 15 participants before and after the consumption of deep-fried foods was conducted as a proof of concept to identify MACs associated with exposure to reactive chemical species derived from deep-fried foods. Detailed information for sample preparation, UHPLC–HRMS analysis, candidate filtering, and structural identification in the deep-fried foods study is available in the Supporting Information “Experimental Details of the Deep-Fried Foods Study” section.

Using the developed urinary MAC profiling strategy, 847 features were mapped to at least one MAC structure in urine samples from 15 participants. These mapped features represented 624 unique structures, including 581 MACs not recorded in either HMDB or PubChem structural databases. The process of structural annotation based on SIRIUS software was illustrated using the MAC of a phase I metabolite of 4-hydroxydecenal as an example (Figure 5). First, the DIA MS/MS spectrum of the MAC candidate was input into the software (Figure 5a). Using the precursor ion and fragment *m*/*z* information, a fragmentation tree was constructed for each possible molecular formula of the precursor ion (Figure 5b). The top 10 potential molecular formulas were ranked based on their calculated similarity measures. These similarity measures were then matched against the molecular fingerprints of chemical structures in the input databases, which included the generated MAC structural database, HMDB, and PubChem structural database. The top-ranked result, the MAC of a phase I metabolite of 4-hydroxydecenal, was considered the final mapping result (Figure 5c). Detailed information on the 847 features mapped with corresponding MAC structures was provided in Supporting Information Table S4. According to the confidence level system established by Schymanski et al.,^58^ identification where evidence exists for possible structures but lacks sufficient information for one exact structure only should be assigned as level 3 tentative candidates. Accordingly, the 847 mapped features were initially assigned confidence level 3 as tentative candidates. All mapped features, regardless of whether their candidate structures were present in HMDB/PubChem or only in the in-house MAC structural database, were annotated using the same in silico workflow and were assigned as level 3 tentative candidates unless verified by authentic standards. Therefore, although the in-house database improves annotation coverage, it does not eliminate the possibility of false-positive or isomeric assignments. Two of these candidates were subsequently upgraded to level 1 through authentic-standard confirmation, namely *N*-acetyl-S-(3-carboxy-2-propyl)-l-cysteine and *N*-acetyl-S-propyl-l-cysteine. In addition, several entries in Table S4 were annotated with the same MAC structure or parent compound name. This may result from (1) the presence of isomeric compounds that are not distinguishable based on UHPLC-HRMS data alone or (2) the formation of multiple distinct MACs derived from a single parent compound through alternative conjugation or biotransformation pathways during the construction of the MAC structural database, which can be distinguished based on the “SMILES of MAC” column.

**Figure 5:**
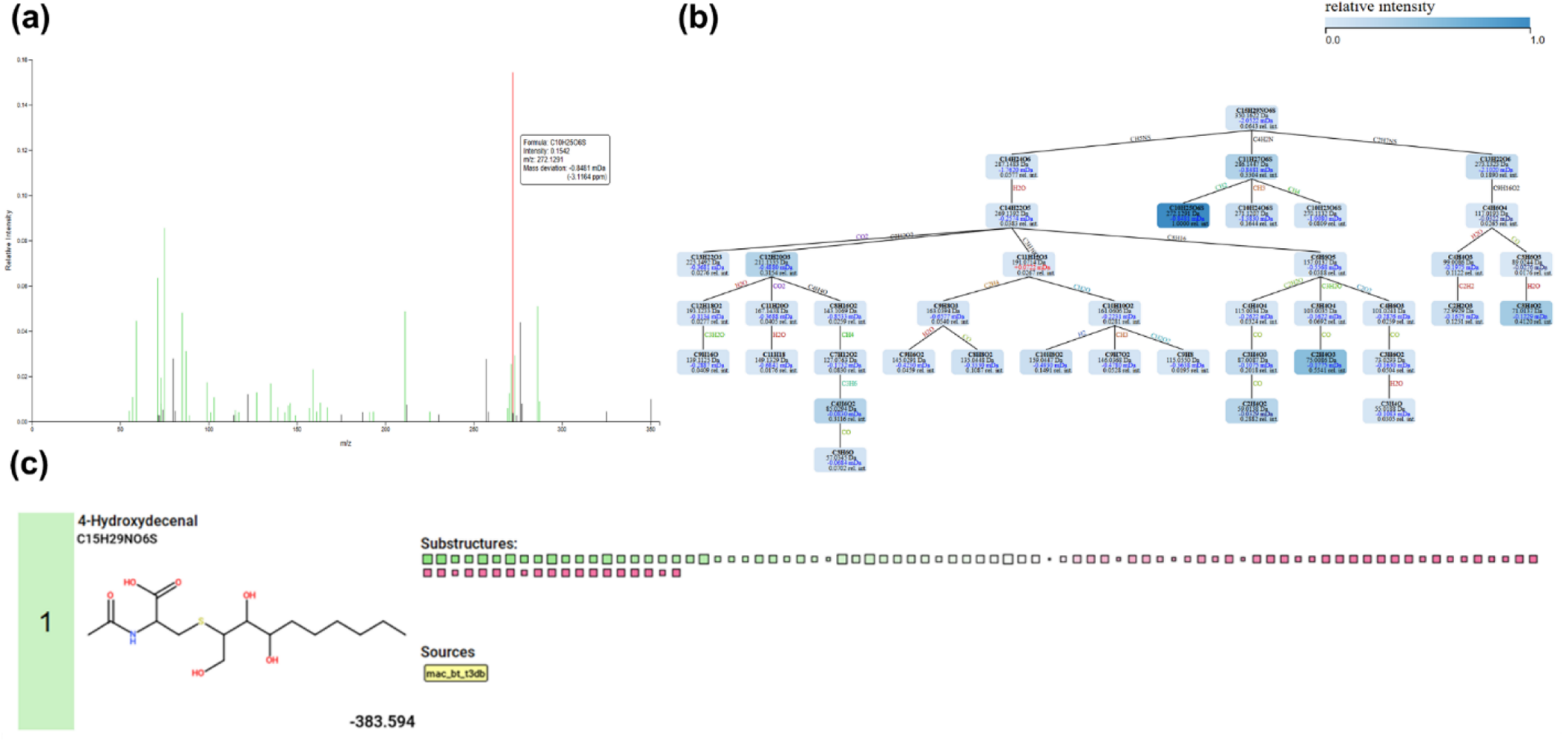
Example of SIRIUS annotation results for the MAC of a phase I metabolite of 4-hydroxydecenal (a) The DIA MS/MS spectrum of the MAC derived from the phase I metabolite of 4-hydroxydecenal. Green-labeled fragments indicate substructures for which at least one molecular formula can be annotated. (b) The fragmentation tree of the MAC. (c) The top-ranked result, determined by matching the similarity measure and molecular fingerprint with the MAC structures in the database.

To further validate the reliability of the nontargeted screening results and enhance the identification confidence of the tentative candidates (level 3), five authentic MAC standards were selected for chromatographic and mass spectrometric confirmation. These selected standards included *N*-acetyl-S-(3-carboxy-2-propyl)-l-cysteine, *N*-acetyl-S-(3-hydroxypropyl)-l-cysteine, *N*-acetyl-S-propyl-l-cysteine, *N*-acetyl-S-(*N*-methylcarbamoyl)-l-cysteine, and *N*-acetyl-S-(4-hydroxy-2-buten-1-yl)-l-cysteine. Through a rigorous comparison of retention times and MS/MS fragmentation patterns between these authentic standards and the experimental urine sample data, *N*-acetyl-S-(3-carboxy-2-propyl)-l-cysteine and *N*-acetyl-S-propyl-l-cysteine were unambiguously identified in the biological samples (Supporting Information Figures S5 and S6). To faithfully represent the raw data pipeline, the top-ranked structures predicted by SIRIUS software were also provided. While top-ranked computational predictions are not perfectly identical to the correct structure, they consistently exhibit high structural similarity to the true standard compounds. Furthermore, a comparative evaluation of the structural annotation results revealed that the features predicted by the SIRIUS software and the in-house MAC structural database were constitutional isomers of the actual verified standards. This observation highlights the capability of the *in silico* tools to accurately capture the core molecular scaffold while simultaneously reinforcing the necessity of authentic standard verification to resolve isomeric ambiguity and achieve definitive structural confirmation.

Compared with previous nontargeted MAC profiling studies, the strategy developed in this work substantially increased both the number of filtered MAC candidates and the number of structurally annotated MACs. Specifically, the total number of urinary MACs annotated in this study was approximately 7-fold higher than the 116 MACs reported by Xie et al.^31^ and approximately 6-fold higher than the 146 MACs reported by Chen et al.^32^ Although Murray et al. and Jamin et al.^30,34^ did not report the specific number of all MACs annotated in urine, they documented between 82 and 1,043 candidate features based on characteristic neutral loss and fragment ion patterns. In contrast, by integration of an additional screening layer based on enzymatic deacetylation, the current approach increased the number of filtered MAC candidates to 4,126. The incorporation of this orthogonal strategy, together with the development of a dedicated MAC structural database, offers complementary advantages to those of existing workflows and may support broader coverage in MAC profiling.

To investigate MACs with significant changes before and after deep-fried food consumption, annotated MACs were filtered based on significant increases or decreases in abundance between urine samples collected during weeks in normal diet and deep-fried food consumption. Urine samples collected on the fourth and fifth day were analyzed separately to minimize variability and reduce the risk of losing significant differential signals that might occur if data from both days were combined. Among the 847 features mapped to MACs, 41 and 18 features showed differential increases based on the exploratory criteria (paired Student’s *t* test, *p*-value <0.05, and fold change >2) on the fourth and fifth day, respectively. Conversely, 4 and 7 features showed significant decreases (paired Student’s *t* test, p-value <0.05, and fold change <0.5) on the fourth and fifth day, respectively. The volcano plots illustrating the distribution of fold changes and paired *t* test, *p*-values for the 847 MACs on the fourth and fifth day are presented in Figure 6. To address multiple testing across the 847 mapped MAC features, Benjamini–Hochberg-adjusted *q*-values were additionally calculated separately for the fourth-day and fifth-day comparisons using the paired Student’s *t* test *p*-values. These *q*-values were reported together with the uncorrected *p*-values in Supporting Information Table S4. Because this proof-of-concept study was designed for exploratory candidate prioritization rather than confirmatory biomarker validation, MACs with differential abundance were still selected using the uncorrected paired Student’s *t* test *p*-value <0.05 combined with fold-change thresholds. Therefore, candidates meeting the uncorrected *p*-value and fold-change criteria should be interpreted as exploratory differential MAC signals, while the *q*-values provide additional information regarding their reliability after multiple-testing correction. A detailed list of MACs with significant changes between deep-fried food consumption and normal-diet conditions is provided in Table 2 (the fourth day) and Table 3 (the fifth day). Compound names in Tables 2 and 3 represent the possible parent compounds of the annotated MACs. The structural formulas of these MACs are listed in Supporting Information Table S5 and Table S6. Cohen’s d values and 95% confidence intervals for all MACs with significant abundance changes are illustrated in Supporting Information Figures S7–S9. It is noted that this study adopted a significance threshold of *p* < 0.05 ^31,59^ to identify MACs potentially associated with deep-fried food consumption. Further investigations are required to elucidate the causal relationship between MAC levels and dietary intake of deep-fried foods.

**Figure 6:**
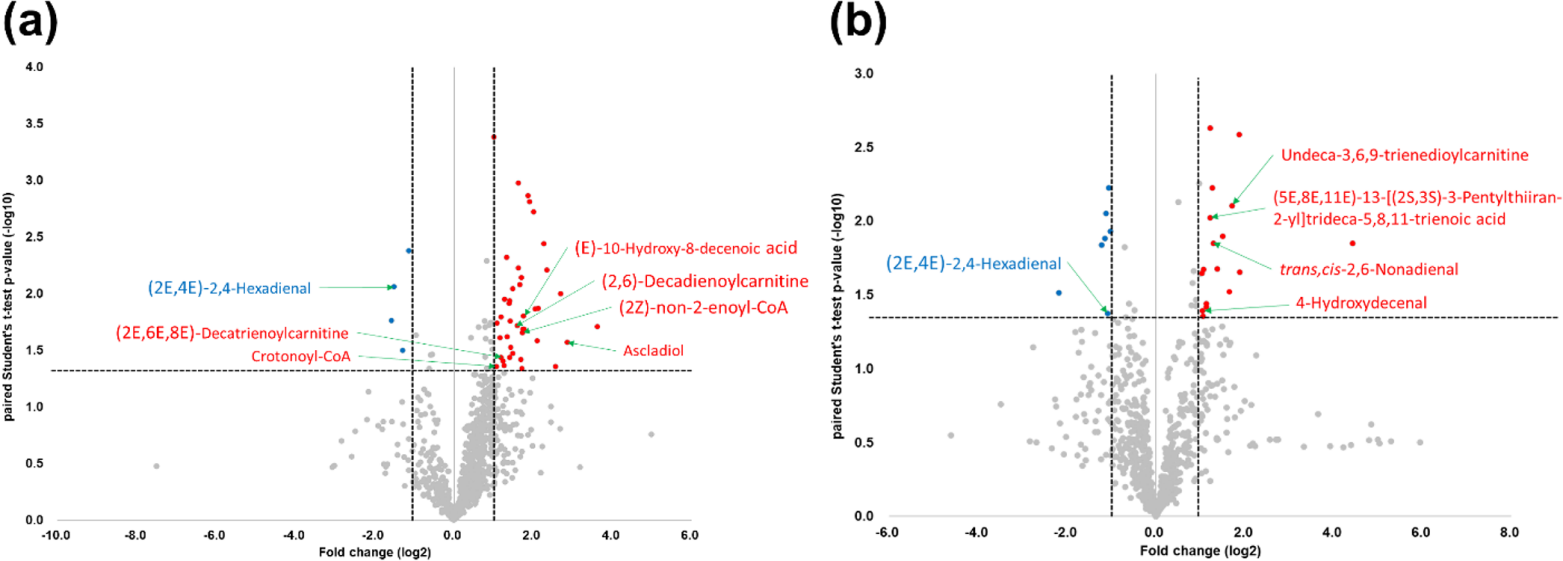
Volcano plots showing differences in abundances of 847 features mapped with MACs between deep-fried food consumption and normal-diet conditions on (a) the fourth day and (b) the fifth day. The two-tailed paired Student’s *t* test was conducted to examine the mean differences of the abundance of 847 features mapped with MACs between deep-fried food consumption and normal-diet conditions. Red points represent *p*-value <0.05 and fold change >2. Blue points represent *p*-value <0.05 and fold change <0.5. Gray points represent *p*-value >0.05 or 0.5 < fold change <2.

**Table 2:** MACs with Significant Changes on the Fourth Day

*m/z*	RT (min)	Expected parent compounds of mapped MACs^a^	Molecular formula of MACs	Fold change (log_2_)	Paired *t* test *p*-value
422.1853	6.5	1,1′-Azobis(cyclohexanecarbonitrile);1,1′-Diazene-1,2-diyldicyclohexanecarbonitrile	C_19_H_29_N_5_O_4_S	3.6	0.020
318.0654	3.3	Ascladiol	C_12_H_17_NO_7_S	2.9	0.027
461.1670	4.5	Ethyl 3-[[(4-chlorophenyl)methyl-[(5-nitrothiophen-2-yl)methyl]amino]methyl]pyrrolidine-1-carboxylate	C_22_H_30_N_4_O_3_S_2_	2.7	0.010
429.1199	6.3	2-Deoxy-2,3-dehydro-*n*-acetyl-neuraminic acid	C_14_H_26_N_2_O_11_S	2.6	0.044
311.0417	1.0	Thiocyanic acid, *p*-aminophenyl ester	C_12_H_14_N_3_O_3_S_2_	2.3	0.006
335.1620	6.2	Isometheptene	C_14_H_28_N_2_O_5_S	2.3	0.004
254.0863	1.9	(3E,5Z)-1,3,5-Heptatriene	C_12_H_17_NO_3_S	2.1	0.013
461.1665	4.6	Ethyl 3-[[(4-chlorophenyl)methyl-[(5-nitrothiophen-2-yl)methyl]amino]methyl]pyrrolidine-1-carboxylate	C_22_H_30_N_4_O_3_S_2_	2.1	0.026
350.1627	7.1	4-Hydroxydecenal	C_15_H_29_NO_6_S	2.1	0.014
402.1956	7.4	(2E,4E)-*N*-[2-[[(2R,3R,4R,5R,6S)-2-(1,2-Dihydroxyethyl)-4,5-dihydroxy-6-(7H-purin-6-ylamino)oxan-3-yl]amino]-2-oxoethyl]tetradeca-2,4-dienamide;(2E,4E)-Tetradecadienoylcarnitine	C_19_H_33_NO_6_S	2.0	0.002
366.1010	6.0	6-(3,4-Methylenedioxyphenyl)-3,5-hexadien-2-one	C_17_H_21_NO_6_S	1.9	0.002
332.1539	6.2	3-Decen-2-one	C_15_H_27_NO_5_S	1.9	0.001
358.0596	4.9	*N*-(Cyclopropylmethoxy)-*N*-(3,5-dichloropyridin-4-yl)-4-(difluoromethoxy)benzamide	C_14_H_18_ClN_3_O_4_S	1.8	0.021
380.1356	5.6	(E)-10-Hydroxy-8-decenoic acid	C_15_H_27_NO_8_S	1.7	0.016
290.1434	5.4	(E)-2-Octene;(Z)-2-Octene	C_13_H_25_NO_4_S	1.7	0.021
330.1386	6.4	(2,6)-Decadienoylcarnitine	C_15_H_25_NO_5_S	1.7	0.022
364.1435	5.2	Diaminopropane	C_15_H_27_NO_7_S	1.7	0.046
362.1648	6.6	2-Undecenal	C_16_H_29_NO_6_S	1.7	0.007
374.1437	6.2	Lactarofulvene	C_20_H_25_NO_4_S	1.7	0.038
367.0784	8.2	1-Isothiocyanato-6-(methylsulfinyl)hexane;1-Isothiocyanato-6-(methylthio)hexane	C_13_H_24_N_2_O_4_S_3_	1.7	0.008
290.1435	5.5	(E)-2-Octene;(Z)-2-Octene	C_13_H_25_NO_4_S	1.6	0.006
332.1539	6.1	3-Decen-2-one	C_15_H_27_NO_5_S	1.6	0.001
377.1574	7.6	(2Z)-non-2-enoyl-CoA	C_16_H_30_N_2_O_4_S_2_	1.6	0.019
343.9696	5.9	1,1,2,2,3,3-Hexachlorocyclohexane	C_11_H_14_Cl_3_NO_3_S	1.5	0.009
593.1918	4.9	rac Benidipine hydrochloride	C_26_H_34_N_4_O_10_S	1.5	0.034
234.0439	2.0	3-Bromo-1-propanol;3-Chloro-1-propanol;Allyl 3-chloro-2-hydroxypropyl ether;Mefenorex	C_8_H_13_NO_5_S	1.4	0.030
307.1127	5.9	2-Pentylpyridine	C_15_H_20_N_2_O_3_S	1.4	0.017
234.0444	2.1	3-Bromo-1-propanol;3-Chloro-1-propanol;Allyl 3-chloro-2-hydroxypropyl ether;Mefenorex	C_8_H_13_NO_5_S	1.4	0.036
333.1568	5.5	3-{[3-(Dimethylamino)propyl]amino}propanenitrile	C_13_H_26_N_4_O_4_S	1.4	0.012
259.0755	5.2	(2Z)-2-Pentenenitrile	C_10_H_16_N_2_O_4_S	1.4	0.012
290.1433	7.0	1-Bromooctane;1-Chlorooctane;1-Fluorooctane	C_13_H_25_NO_4_S	1.3	0.024
356.1355	7.5	Lactarofulvene	C_20_H_23_NO_3_S	1.3	0.005
315.0849	5.9	7-Isothiocyanato-1-heptene	C_13_H_20_N_2_O_3_S_2_	1.3	0.011
387.0931	4.7	4-Methoxy-3-nitro-*N*-phenylbenzamide	C_18_H_18_N_3_O_5_S	1.3	0.043
306.1018	4.2	*tert*-Butyl acrylate	C_12_H_21_NO_6_S	1.2	0.040
328.1224	6.9	(2E,6E,8E)-Decatrienoylcarnitine	C_15_H_23_NO_5_S	1.2	0.037
364.1441	5.0	Diaminopropane	C_15_H_27_NO_7_S	1.2	0.016
318.1384	6.0	3-Nonen-2-one	C_14_H_25_NO_5_S	1.2	0.024
243.0442	2.7	Dihydromaleimide beta-d-glucoside	C_9_H_12_N_2_O_4_S	1.1	0.018
379.1027	6.8	(2E)-Butenoyl-CoA;Crotonoyl-CoA	C_14_H_24_N_2_O_6_S_2_	1.1	0.044
304.1593	7.9	1-Chlorononane;1-Iodononane;*n*-Nonyl bromide	C_14_H_27_NO_4_S	1.0	<0.001
325.0947	0.9	Lazabemide hydrochloride	C_13_H_18_N_4_O_4_S	–1.1	0.004
354.0931	0.9	Carabersat	C_15_H_19_N_2_O_6_S	–1.3	0.032
290.0690	1.0	(2E,4E)-2,4-Hexadienal;(E,E)-2,4-Hexadienal	C_11_H_17_NO_6_S	–1.5	0.009
242.0105	0.9	1,1,2-Trichloro-1,2,2-trifluoroethane;Chloropentafluoroethane;Chlorotrifluoroethylene	C_7_H_8_F_3_NO_3_S	–1.6	0.017

a When MAC is associated with multiple parent compounds, separate parent compounds are used with semicolons.

**Table 3:** MACs with Significant Changes on the Fifth Day

*m/z*	RT (min)	Expected parent compounds of mapped MACs^a^	Molecular formula of MACs	Fold change (log_2_)	Paired *t* test *p-*value
363.1928	7.0	3-(Octyloxy)propan-1-amine	C_16_H_32_N_2_O_5_S	4.4	0.014
332.1268	5.0	*N*-(2-Cyanoethyl)valine	C_13_H_20_N_3_O_5_S	2.4	0.017
445.0787	6.2	Triflumizole	C_17_H_17_F_3_N_4_O_5_S	1.9	0.022
382.0969	6.2	6-(3,4-Methylenedioxyphenyl)-3,5-hexadien-2-one	C_17_H_21_NO_7_S	1.9	0.003
372.1117	5.7	Undeca-3,6,9-trienedioylcarnitine	C_16_H_23_NO_7_S	1.7	0.008
513.0652	6.1	Diclosulam	C_18_H_19_FN_6_O_7_S_2_	1.6	0.030
296.0963	6.1	Violet-leaf aldehyde;trans,cis-2,6-Nonadienal	C_14_H_19_NO_4_S	1.5	0.013
260.0326	3.3	(3-Chlorophenyl)hydrazonomalononitrile;Carbonyl cyanide chlorophenylhydrazone;Carbonyl cyanide *m*-chlorophenyl hydrazone;Carbonyl cyanide p-trifluoromethoxyphenylhydrazone;Carbonyl cyanide-p-trifluoromethoxyphenylhydrazone;Levosimendan;Mesoxalonitrile	C_8_H_11_N_3_O_5_S	1.4	0.021
442.1538	6.0	Ovalicin	C_20_H_29_NO_8_S	1.3	0.014
295.0667	1.0	Nitroxinil	C_12_H_14_N_3_O_4_S	1.3	0.006
442.1539	5.8	Ovalicin	C_20_H_29_NO_8_S	1.2	0.002
494.2032	6.5	(5E,8E,11E)-13-[(2*S*,3*S*)-3-Pentylthiiran-2-yl]trideca-5,8,11-trienoic acid	C_25_H_37_NO_5_S_2_	1.2	0.010
388.1606	5.1	2,6-Diisopropylnaphthalene	C_21_H_27_NO_4_S	1.1	0.036
383.1895	7.7	Chlorazine	C_16_H_28_N_6_O_3_S	1.1	0.038
358.0608	5.1	5-Chloro-3-ethyl-*N*-[(1-ethylpyrrolidin-2-yl)methyl]-2-hydroxy-6-methoxybenzamide;Eticlopride hydrochloride	C_14_H_17_NO_8_S	1.1	0.021
325.0947	0.9	Lazabemide hydrochloride	C_13_H_18_N_4_O_4_S	1.1	0.044
350.1627	7.1	4-Hydroxydecenal	C_15_H_29_NO_6_S	1.0	0.040
377.0546	3.8	(2S)-2-Amino-3-[4-[(2S)-2-amino-3-(4-hydroxyphenyl)propanoyl]oxy-3-chlorophenyl]propanoic acid	C_14_H_19_ClN_2_O_6_S	1.0	0.023
295.0450	1.1	Homocysteine	C_9_H_16_N_2_O_5_S_2_	–1.0	0.012
349.0645	1.1	Thiocarboxime	C_11_H_18_N_4_O_5_S_2_	–1.1	0.006
290.0690	1.0	(2E,4E)-2,4-Hexadienal;(E,E)-2,4-Hexadienal	C_11_H_17_NO_6_S	–1.1	0.042
309.0909	6.7	Phenethyl isocyanate	C_14_H_18_N_2_O_4_S	–1.1	0.009
340.0574	6.6	3-Carbamyl-(3′-picolyl)-4-methoxy-1-benzamide	C_13_H_15_N_3_O_6_S	–1.1	0.013
295.0447	1.4	Homocysteine	C_9_H_16_N_2_O_5_S_2_	–1.2	0.014
190.0176	1.1	Formic acid	C_6_H_9_NO_4_S	–2.2	0.031

a When MAC is associated with multiple parent compounds, separate parent compounds with semicolons.

Reactive aldehydes constituted a major class of MACs that significantly increased following deep-fried food consumption. These electrophiles are primarily generated via the peroxidative degradation of unsaturated fatty acids during frying.^35,60,61^ Several aldehyde-derived MACs, including those originating from 2-undecenal, 4-hydroxydecenal, and 2,6-nonadienal, showed marked postexposure increases in urine. Such aldehydes are commonly detected in fried oils and are characterized by their high electrophilic reactivity.^62,63^ In addition to aldehydes, MACs derived from other lipid oxidation products—such as 3-decen-2-one, 2-pentenenitrile, and 10-hydroxy-8-decenoic acid—were also elevated, consistent with their formation through thermal oxidation of lipids or high-temperature degradation of amino acids and fatty acids. Notably, these compounds represent significant systemic exposures to reactive electrophiles; for example, 2-pentenenitrile is a known reactive species generated during high-temperature cooking.^64,65^ Beyond exogenous lipid-derived compounds, several MACs associated with acylcarnitines and acyl-CoA intermediates, including decadienoylcarnitine and crotonoyl-CoA, are significantly increased. Unlike frying-oil-derived compounds, these metabolites are endogenous intermediates of mitochondrial fatty acid β-oxidation.^66,67^ Their elevation may therefore reflect perturbations in lipid metabolism following dietary exposure, consistent with previous reports linking similar metabolites to mitochondrial dysfunction.^68^ Importantly, as this study is primarily at the exposure biomarker discovery stage, these identified MACs serve as potential risk indicators of reactive chemical species exposure rather than diagnostic biomarkers for specific clinical diseases. Their predictive value and clinical relevance will require validation through subsequent dose–response and epidemiological studies.

A subset of increased MACs was annotated as originating from synthetic drugs, pesticides, or industrial chemicals. However, because direct evidence for the presence of these specific parent compounds in the consumed deep-fried foods is lacking, these unexpected annotations likely reflect two alternative possibilities rather than direct external exposure. First, these compounds may represent structural analogs or isomeric species generated during high-temperature cooking. For example, although ascladiol is a mycotoxin and unlikely to be directly present in deep-fried foods, it shares structural features with furanyl-containing compounds formed via lipid oxidation or Maillard reactions during high-temperature cooking.^69,70^ Second, these MACs could equally originate from endogenous reactive intermediates produced due to metabolic imbalances triggered by the high dietary intake of deep-fried foods. For instance, the acute influx of dietary oxidized lipids can induce systemic metabolic dysregulation and lipid accumulation. These accumulating lipids may undergo lipid peroxidation triggered by cellular oxidants, thereby generating potent endogenous electrophiles. These resulting endogenous reactive species—such as electrophilic α,β-unsaturated aldehydes and oxidized phospholipids—would subsequently undergo glutathione conjugation for detoxification, ultimately being excreted as urinary MACs.^71^ Other similar biochemical reactions may also occur in the body, generating the MACs of the structural analogs of some artificially synthesized compounds. Conversely, several MACs, including those derived from 2,4-hexadienal, showed significant decreases after exposure, potentially due to competitive detoxification among multiple electrophiles.^72^

The limited overlap of significantly altered mercapturic acids (MACs) between the fourth and fifth day is likely attributable to differences in the metabolic and elimination kinetics of the involved xenobiotics. On the fourth day, urine samples were collected shortly after deep-fried food consumption, capturing the rapid excretion of electrophiles with short biological half-lives. For example, ethyl 3-[[(4-chlorophenyl)­methyl-[(5-nitrothiophen-2-yl)­methyl]­amino]­methyl]­pyrrolidine-1-carboxylate, which has a reported half-life of approximately 2 h,^73^ was promptly detected postexposure. In contrast, sampling on the fifth day followed exposure cessation, and the observed MACs likely reflected biotransformation products of more persistent xenobiotics that accumulated during repeated intake. This is exemplified by *N*-(2-cyanoethyl)­valine, which has an estimated half-life of ∼30 days and may remain detectable after exposure ends.^74^ These temporal differences in xenobiotic clearance plausibly contributed to the observed day-specific MAC profiles.

False-positive results might exist when using aminoacylase-1-mediated deacetylation as the sole criterion for screening MACs due to the enzyme’s broad substrate specificity. According to UniProt, aminoacylase-1 (Q03154) also catalyzes the hydrolysis of various non-MAC N-acetylated compounds, such as *N*-acetyl-l-glutamine (Rhea ID: 67368), which may lead to the misclassification of unrelated metabolites as MACs based on their signal reduction after enzymatic treatment. For instance, two features from the deep-fried food dataset (#6119 and #14102) initially showed decreased intensities and were flagged as MAC candidates but were subsequently identified, through spectral matching against MoNA and structure prediction using SIRIUS, as isomers of *N*-acetylasparagine and *N*-acetyl-O-methyltyrosine, respectively. These findings indicate that aminoacylase-1-mediated deacetylation should be regarded as a screening criterion rather than definitive structural evidence for MAC identification, and they highlight the importance of integrating structure-based confirmation to accurately distinguish true MACs from nonspecific deacetylation products. Comprehensive quantification of the false-positive proportion would require authentic standards or orthogonal structural confirmation for a large number of decreased urinary features, which is not feasible in the present nontargeted study. Therefore, all candidates were conservatively reported as tentative annotations unless confirmed by authentic standards.

Despite the improved coverage achieved in this study, several methodological limitations must be acknowledged. First, the enzymatic deacetylation step relies on aminoacylase-1, which exhibits broad substrate specificity and may introduce nonspecific transformations, necessitating careful downstream structural validation. Second, structural annotation remains largely at confidence level 3 due to the limited availability of authentic standards and the inherent ambiguity in distinguishing structural isomers using MS/MS data alone. Future studies should include larger panels of authentic MAC standards with broader structural diversity, including heterocyclic, aliphatic diene-containing, dithiocarbamate-related, and other labile MACs, to verify more identified MACs and systematically evaluate subclass-dependent differences in enzymatic deacetylation efficiency and neutral loss behavior. Third, the reliance on a large in silico database, while expanding coverage, may increase the risk of false-positive annotations without orthogonal confirmation. There are also several methodological limitations in the deep-fried food study. The participants had a relatively small sample size (*n* = 15) and an unbalanced gender ratio (11 males and 4 females). Because this application was primarily designed as a proof-of-concept to demonstrate the analytical feasibility of the developed MAC identification workflow, participant recruitment was localized to our research institute to optimize sampling convenience and collection costs. In addition, detailed preparation conditions for the distributed deep-fried foods were not recorded in sufficient detail for source-specific exposure reconstruction. Consequently, these demographic and exposure characterization limitations may affect the generalizability and interpretability of the specific biological results. Future large-scale studies with diverse and balanced demographics, together with better-standardized dietary preparation metadata, are required to fully elucidate the biological impacts and predictive value of these exposure biomarkers in relation to the dietary intake of deep-fried foods.

Future improvements could be achieved by integrating complementary analytical strategies, such as ion mobility spectrometry to enhance isomer separation, targeted MS/MS acquisition (e.g., parallel reaction monitoring) for higher-confidence validation, and stable isotope labeling approaches, to improve quantitative reliability. In addition, coupling this workflow with machine-learning-assisted spectral prediction and retention time modeling may further refine structural annotation accuracy. Such multiplatform integration is expected to enhance both the selectivity and confidence of MAC profiling in complex biological matrices.

## Conclusion

In summary, we developed and applied a comprehensive strategy for profiling human urinary MACs by integrating enzyme deacetylation with HRMS-DIA-based neutral-loss filtering to discover MACs related to deep-fried food consumption. The performance of the strategy in MAC candidate filtering and structure annotation was validated by using 11 MAC standards. In the deep-fried food study, 847 features were structurally mapped to at least one MAC structure in urine samples collected from 15 participants. Among these, elevated MACs were linked to aldehydes, lipid oxidation products, and intermediates of acylcarnitine and acyl-CoA metabolism. These altered MACs could be interpreted as candidate exposure or risk indicators of reactive chemical species, and their predictive value requires validation in future dose–response and epidemiological studies. The developed strategy provides an analytical framework that can be further expanded to profile a broader spectrum of MACs across a diverse set of samples.

## Supplementary Material





## Notes

Experimental details of the deep-fried foods study; demographics of the 15 participants in this study (Table S1); list of 11 MAC standards with their structures (Table S2); structures and structural elucidation results of MPhMA, 5HPnFuMA, and 4MSfBAToMMA (Table S3); MACs with significant changes on the fourth day (with structural formulas) (Table S5); MACs with significant changes on the fifth day (with structural formulas) (Table S6); efficiency of aminoacylase-1 in removing acetyl groups from 11 MAC standards under different amounts of aminoacylase-1 (Figure S1); efficiency of aminoacylase-1 in removing acetyl groups from 11 MAC standards under different incubation times (Figure S2); comparison of features detected using various isolation window modes and AIF analysis (Figure S3); MS/MS spectra of chloramphenicol under different isolation windows (Figure S4); comparison of *N*-acetyl-S-(3-carboxy-2-propyl)-l-cysteine in the standard and urine samples (Figure S5); comparison of *N*-acetyl-S-propyl-l-cysteine in the standard and urine samples (Figure S6); Cohen’s d values of all identified MACs with significant abundance changes between urine samples collected under deep-fried food consumption and control conditions (Figure S7); 95% confidence intervals of each identified MAC with significant abundance changes between urine samples collected under deep-fried food consumption and control conditions on day 4 (Figure S8); 95% confidence intervals of each identified MAC with significant abundance changes between urine samples collected under deep-fried food consumption and control conditions on day 5 (Figure S9); MAC filtering and structure identification process of the deep-fried foods study (Figure S10) (PDF)
List of 847 features mapped to at least 1 MAC structure in the urine samples (Table S4) (XLSX)

## References

[1] Idle J. R., Gonzalez F. J. (2007). Metabolomics. Cell Metab..

[2] Štefanac T., Grgas D., Dragičević T. L. (2021). Xenobiotics—Division
and Methods of Detection: A Review. J. Xenobiot..

[3] Donner, E. ; Eriksson, E. ; Holten-Lützhøft, H.-C. ; Scholes, L. ; Revitt, M. ; Ledin, A. Identifying and Classifying the Sources and Uses of Xenobiotics in Urban Environments. In Xenobiotics in the Urban Water Cycle: mass Flows, Environmental Processes, Mitigation and Treatment Strategies, Fatta-Kassinos, D. ; Bester, K. ; Kümmerer, K. , Eds., Springer: Springer Netherlands, 2010, pp. 27–50.

[4] Halliwell B., Whiteman M. (2004). Measuring reactive
species and oxidative damage in
vivo and in cell culture: how should you do it and what do the results
mean?. Br. J. Pharmacol..

[5] Fleming E., Luo Y. (2021). Co-delivery of synergistic
antioxidants from food sources for the
prevention of oxidative stress. J. Agric. Food
Res..

[6] LoPachin R. M., DeCaprio A. P. (2005). Protein Adduct Formation
as a Molecular Mechanism in
Neurotoxicity. Toxicol. Sci..

[7] Balbo S., Turesky R. J., Villalta P. W. (2014). DNA Adductomics. Chem. Res. Toxicol..

[8] Yan L. L., Zaher H. S. (2019). How do cells cope
with RNA damage and its consequences?. J. Biol.
Chem..

[9] De
Flora S., Izzotti A., Randerath K., Randerath E., Bartsch H., Nair J., Balansky R., van Schooten F., Degan P., Fronza G., Walsh D., Lewtas J. (1996). DNA adducts and chronic degenerative diseases. Pathogenetic
relevance and implications in preventive medicine. Mutat. Res., Rev. Genet. Toxicol..

[10] Hwa
Yun B., Guo J., Bellamri M., Turesky R. J. (2020). DNA adducts: Formation,
biological effects, and new biospecimens for mass spectrometric measurements
in humans. Mass Spectrom. Rev..

[11] Ketterer B., Coles B., Meyer D. J. (1983). The role
of glutathione in detoxication. Environ. Health
Perspect..

[12] Shen G., Kong A. N. (2009). Nrf2 plays an important
role in coordinated regulation
of Phase II drug metabolism enzymes and Phase III drug transporters. Biopharm. Drug Dispos..

[13] Iyanagi T. (2007). Molecular
Mechanism of Phase I and Phase II Drug-Metabolizing Enzymes: Implications
for Detoxification. Int. Rev. Cytol..

[14] Marnett L. J., Riggins J. N., West J. D. (2003). Endogenous
generation of reactive
oxidants and electrophiles and their reactions with DNA and protein. J. Clin. Invest..

[15] Cooper, A. J. L. ; Hanigan, M. H. Enzymes Involved in Processing Glutathione Conjugates. In Comprehensive Toxicology, 2nd, ed., McQueen, C. A. , ed., Elsevier: Oxford, 2010, pp. 323–366.

[16] Seutter-Berlage F., van Dorp H. L., Kosse H. G. J., Henderson P. T. (1977). Urinary
mercapturic acid excretion as a biological parameter of exposure to
alkylating agents. Int. Arch. Occup. Environ.
Health.

[17] Weisel C. P. (2010). Benzene
exposure: An overview of monitoring methods and their findings. Chem.-Biol. Interact..

[18] Hoet P., De Smedt E., Ferrari M., Imbriani M., Maestri L., Negri S., De Wilde P., Lison D., Haufroid V. (2009). Evaluation
of urinary biomarkers of exposure to benzene: correlation with blood
benzene and influence of confounding factors. Int. Arch. Occup. Environ. Health.

[19] Wagner S., Scholz K., Donegan M., Burton L., Wingate J., Völkel W. (2006). Metabonomics
and biomarker discovery: LC–MS
metabolic profiling and constant neutral loss scanning combined with
multivariate data analysis for mercapturic acid analysis. Anal. Chem..

[20] Scholz K., Dekant W., Völkel W., Pähler A. (2005). Rapid detection
and identification of N-acetyl-L-cysteine thioethers using constant
neutral loss and theoretical multiple reaction monitoring combined
with enhanced product-ion scans on a linear ion trap mass spectrometer. J. Am. Soc. Mass Spectrom..

[21] Xian F., Hendrickson C. L., Marshall A. G. (2012). High Resolution Mass Spectrometry. Anal. Chem..

[22] Marshall A. G., Hendrickson C. L. (2008). High-Resolution Mass Spectrometers. Annu. Rev. Anal. Chem..

[23] Chen Y. C., Hsu J. F., Chang C. W., Li S. W., Yang Y. C., Chao M. R., Chen H. J. C., Liao P. C. (2023). Connecting chemical
exposome to human health using high-resolution mass spectrometry-based
biomonitoring: Recent advances and future perspectives. Mass Spectrom. Rev..

[24] Bloszies C. S., Fiehn O. (2018). Using untargeted metabolomics
for detecting exposome compounds. Curr. Opin.
Toxicol..

[25] Wang R., Yin Y., Zhu Z.-J. (2019). Advancing untargeted metabolomics using data-independent
acquisition mass spectrometry technology. Anal.
Bioanal. Chem..

[26] Bonner R., Hopfgartner G. (2019). SWATH data
independent acquisition mass spectrometry
for metabolomics. TrAC, Trends Anal. Chem..

[27] Dai W., Yin P., Zeng Z., Kong H., Tong H., Xu Z., Lu X., Lehmann R., Xu G. (2014). Nontargeted modification-specific
metabolomics study based on liquid chromatography–high-resolution
mass spectrometry. Anal. Chem..

[28] Chen Y.-C., Wu H.-Y., Chang C.-W., Liao P.-C. (2022). Post-Deconvolution
MS/MS Spectra Extraction with Data-Independent Acquisition for Comprehensive
Profiling of Urinary Glucuronide-Conjugated Metabolome. Anal. Chem..

[29] Debrauwer L., Mervant L., Laprevote O., Jamin E. L. (2025). Pivotal Role of
Mass Spectrometry for the Assessment of Exposure to Reactive Chemical
Contaminants: From the Exposome to the Adductome. Mass Spectrom. Rev..

[30] Jamin E. L., Costantino R., Mervant L., Martin J.-F., Jouanin I., Blas-Y-Estrada F., Guéraud F., Debrauwer L. (2020). Global Profiling
of Toxicologically Relevant Metabolites in Urine: Case Study of Reactive
Aldehydes. Anal. Chem..

[31] Xie Z., Chen J. Y., Gao H., Keith R. J., Bhatnagar A., Lorkiewicz P., Srivastava S. (2023). Global Profiling of Urinary Mercapturic
Acids Using Integrated Library-Guided Analysis. Environ. Sci. Technol..

[32] Chen J. Y., Sutaria S. R., Xie Z., Kulkarni M., Keith R. J., Bhatnagar A., Sears C. G., Lorkiewicz P., Srivastava S. (2025). Simultaneous
profiling of mercapturic acids, glucuronic
acids, and sulfates in human urine. Environ.
Int..

[33] Bloch R., Schütze S.-E., Müller E., Röder S., Lehmann I., Brack W., Krauss M. (2019). Non-targeted mercapturic
acid screening in urine using LC-MS/MS with matrix effect compensation
by postcolumn infusion of internal standard (PCI-IS). Anal. Bioanal. Chem..

[34] Murray K. J., Mckeon D., Lecchi C., Maertens L., Villalta P. W., Balbo S. (2025). Positive Ion Tandem Mass Spectrometry
Offers Enhanced Structural
Insights for the Discovery of Mercapturic Acids. Chem. Res. Toxicol..

[35] Grootveld M., Percival B. C., Leenders J., Wilson P. B. (2020). Potential Adverse
Public Health Effects Afforded by the Ingestion of Dietary Lipid Oxidation
Product Toxins: Significance of Fried Food Sources. Nutrients.

[36] Wang T.-W., Liu J.-H., Tsou H.-H., Liu T.-Y., Wang H.-T. (2019). Identification
of acrolein metabolites in human buccal cells, blood, and urine after
consumption of commercial fried food. Food Sci.
Nutr..

[37] Warrack B. M., Hnatyshyn S., Ott K.-H., Reily M. D., Sanders M., Zhang H., Drexler D. M. (2009). Normalization strategies for metabonomic
analysis of urine samples. J. Chromatogr. B.

[38] Bijlsma S., Bobeldijk I., Verheij E. R., Ramaker R., Kochhar S., Macdonald I. A., van Ommen B., Smilde A. K. (2006). Large-Scale Human
Metabolomics Studies: A Strategy for Data (Pre-) Processing and Validation. Anal. Chem..

[39] Correia M. S., Rao M., Ballet C., Globisch D. (2019). Coupled Enzymatic Treatment and Mass
Spectrometric Analysis for Identification of Glucuronidated Metabolites
in Human Samples. ChemBioChem.

[40] Ballet C., Correia M. S., Conway L. P., Locher T. L., Lehmann L. C., Garg N., Vujasinovic M., Deindl S., Löhr J.-M., Globisch D. (2018). New enzymatic and mass spectrometric methodology for
the selective investigation of gut microbiota-derived metabolites. Chem. Sci..

[41] Comprehensive toxicology, McQueen, C. , ed.; Elsevier, 2017.

[42] Uttamsingh V., Keller D., Anders M. (1998). Acylase I-catalyzed deacetylation
of N-acetyl-L-cysteine and S-alkyl-N-acetyl-L-cysteines. Chem. Res. Toxicol..

[43] Pal V. K., Kannan K. (2024). Stability of volatile
organic compound metabolites
in urine at various storage temperatures and freeze-thaw cycles for
8 months. Environ. Pollut..

[44] Wishart D. S., Guo A., Oler E., Wang F., Anjum A., Peters H., Dizon R., Sayeeda Z., Tian S., Lee B. L. (2022). HMDB 5.0: the human
metabolome database for 2022. Nucleic Acids
Res..

[45] Richard A. M., Huang R., Waidyanatha S., Shinn P., Collins B. J., Thillainadarajah I., Grulke C. M., Williams A. J., Lougee R. R., Judson R. S. (2021). The Tox21 10K compound library: collaborative
chemistry advancing toxicology. Chem. Res. Toxicol..

[46] Wishart D., Arndt D., Pon A., Sajed T., Guo A. C., Djoumbou Y., Knox C., Wilson M., Liang Y., Grant J. (2015). T3DB:
the toxic exposome database. Nucleic Acids Res..

[47] Djoumbou-Feunang Y., Fiamoncini J., Gil-de-la-Fuente A., Greiner R., Manach C., Wishart D. S. (2019). BioTransformer:
a comprehensive computational tool
for small molecule metabolism prediction and metabolite identification. J. Cheminf..

[48] de
Bruyn Kops C., Šícho M., Mazzolari A., Kirchmair J. (2021). GLORYx: prediction of the metabolites resulting from
phase 1 and phase 2 biotransformations of xenobiotics. Chem. Res. Toxicol..

[49] Landrum G. (2013). RDKit documentation. Release.

[50] Chen X., Xia B., Wu W., Jin Z., Wang Y., Zhou Y. (2025). Development
and Application of a Comprehensive Non-targeted Screening Strategy
for Fentanyl Analogues. J. Hazard. Mater..

[51] Wang F., Pasin D., Skinnider M. A., Liigand J., Kleis J.-N., Brown D., Oler E., Sajed T., Gautam V., Harrison S. (2023). Deep learning-enabled
MS/MS spectrum prediction
facilitates automated identification of novel psychoactive substances. Anal. Chem..

[52] Dührkop K., Fleischauer M., Ludwig M., Aksenov A. A., Melnik A. V., Meusel M., Dorrestein P. C., Rousu J., Böcker S. (2019). SIRIUS 4:
a rapid tool for turning tandem mass spectra into metabolite structure
information. Nat. Methods.

[53] Dührkop K., Shen H., Meusel M., Rousu J., Böcker S. (2015). Searching
molecular structure databases with tandem mass spectra using CSI:
FingerID. Proc. Natl. Acad. Sci. U. S. A..

[54] Szabo D., Fischer S., Mathew A. P., Kruve A. (2024). Prioritization, Identification,
and Quantification of Emerging Contaminants in Recycled Textiles Using
Non-Targeted and Suspect Screening Workflows by LC-ESI-HRMS. Anal. Chem..

[55] Vatanen T., Jabbar K. S., Ruohtula T., Honkanen J., Avila-Pacheco J., Siljander H., Stražar M., Oikarinen S., Hyöty H., Ilonen J. (2022). Mobile genetic elements
from the
maternal microbiome shape infant gut microbial assembly and metabolism. Cell.

[56] Crnogorac G., Schwack W. (2007). Determination of dithiocarbamate fungicide residues
by liquid chromatography/mass spectrometry and stable isotope dilution
assay. Rapid Commun. Mass Spectrom..

[57] Chang C., Wu G., Zhang H., Jin Q., Wang X. (2020). Deep-fried flavor:
Characteristics, formation mechanisms, and influencing factors. Crit. Rev. Food Sci. Nutr..

[58] Schymanski E. L., Jeon J., Gulde R., Fenner K., Ruff M., Singer H. P., Hollender J. (2014). Identifying
small molecules via high
resolution mass spectrometry: communicating confidence. Environ. Sci. Technol..

[59] Pathmasiri W., Rushing B. R., McRitchie S., Choudhari M., Du X., Smirnov A., Pelleigrini M., Thompson M. J., Sakaguchi C. A., Nieman D. C. (2024). Untargeted
metabolomics reveal signatures of
a healthy lifestyle. Sci. Rep..

[60] Zamora R., Aguilar I., Granvogl M., Hidalgo F. J. (2016). Toxicologically
relevant aldehydes produced during the frying process are trapped
by food phenolics. J. Agric. Food Chem..

[61] Grootveld M., Percival B. C., Grootveld K. L. (2018). Chronic non-communicable disease
risks presented by lipid oxidation products in fried foods. Hepatobiliary Surg. Nutr..

[62] Girona J., Vallvé J.-C., Ribalta J., Heras M., Olivé S. L., Masana L. S. (2001). 2,4-Decadienal downregulates TNF-α
gene expression
in THP-1 human macrophages. Atherosclerosis.

[63] Cabré A., Girona J., Vallvé J.-C., Heras M., Masana L. X. (2003). Cytotoxic
effects of the lipid peroxidation product 2,4-decadienal in vascular
smooth muscle cells. Atherosclerosis.

[64] Gagnaire F., Marignac B., Ban M., Langlais C. (2001). The Ototoxic Effects
Induced in Rats by Treatment for 12 Weeks with 2-Butenenitrile, 3-Butenenitrile
and cis-2-Pentenenitrile. Pharmacol. Toxicol..

[65] Saldaña-Ruíz S., Hernández-Mir G., Sedó-Cabezón L., Cutillas B., Llorens J. (2012). Vestibular toxicity of cis-2-pentenenitrile in the
rat. Toxicol. Lett..

[66] Reuter S. E., Evans A. M. (2012). Carnitine and acylcarnitines:
pharmacokinetic, pharmacological
and clinical aspects. Clin. Pharmacokinet..

[67] Leonhardt M., Langhans W. (2004). Fatty acid oxidation
and control of food intake. Physiol. Behav..

[68] Miinalainen I. J., Schmitz W., Huotari A., Autio K. J., Soininen R., Ver Loren van Themaat E., Baes M., Herzig K.-H., Conzelmann E., Hiltunen J. K. (2009). Mitochondrial 2,4-dienoyl-CoA reductase
deficiency in mice results in severe hypoglycemia with stress intolerance
and unimpaired ketogenesis. PLoS Genet..

[69] Lykakis I. N., Zaravinos I.-P., Raptis C., Stratakis M. (2009). Divergent
synthesis of the co-isolated mycotoxins longianone, isopatulin, and
(Z)-ascladiol via furan oxidation. J. Org. Chem..

[70] Liu Z., Gao S., Yuan Z., Yang R., Zhang X., El-Mesery H. S., Dai X., Lu W., Xu R. (2025). Exploring Formation and Control of
Hazards in Thermal Processing for Food Safety. Foods.

[71] Ross M. K., Matthews A. T., Mangum L. C. (2014). Chemical atherogenesis: role of endogenous
and exogenous poisons in disease development. Toxics.

[72] Mulder, G. J. Competition between conjugations for the same substrate. In Conjugation Reactions in Drug Metabolism, Mulder, G. J. , ed., CRC Press, 1990, pp. 41–49.

[73] Li H., Song S., Tien C. L., Qi L., Graves A., Nasiotis E., Burris T. P., Zhao Y., Sun Z., Zhang L. (2022). SR9009 improves heart function after pressure overload
independent
of cardiac REV-ERB. Front. Cardiovasc. Med..

[74] Camacho, O. M. ; McEwan, M. ; Gale, N. ; Pluym, N. ; Scherer, M. ; Hardie, G. ; Murphy, J. Use of the Acrylonitrile Haemoglobin Adduct N-(2-cyanoethyl)­valine as a Biomarker Of Compliance in Smokers Switching to Tobacco Heating Products. Preprints. 2021. 10.20944/preprints202108.0085.v1.

