# Supplementary material for: Comprehensive Profiling of Human Urinary Mercapturic Acid Conjugates Associated with Exposure to Reactive Chemical Species Using Enzymatic Deacetylation and High-Resolution Mass Spectrometry
Source: Anal Chem. 2026 Jun 17;98(25):18406–20. doi: 10.1021/acs.analchem.5c08181 (PMC13325442; doi:10.1021/acs.analchem.5c08181)

Supporting Information

Comprehensive Profiling of Human Urinary Mercapturic Acid Conjugates Associated with Exposure to Reactive Chemical Species Using Enzymatic Deacetylation and High-Resolution Mass Spectrometry

Yuan-Chih Chen[1], Man-Ni Zhuang[1], Jen-Yi Hsu[1], Yi-Chun Lin[1], Hsin-Yi Wu[2], and Pao-Chi Liao[1,]*

1. Department of Environmental and Occupational Health, College of Medicine, National Cheng Kung University, Tainan 701, Taiwan

2. Instrumentation Center, National Taiwan University, Taipei 106, Taiwan

*Correspondence:

Dr. Pao-Chi Liao

Department of Environmental and Occupational Health

National Cheng Kung University College of Medicine

138 Sheng-Li Road, Tainan 70428, Taiwan

TEL: 886-6-2353535 ext 5566, FAX: 886-6-2743748

E-mail: liaopc@mail.ncku.edu.tw

Table of contents

Experimental detail of deep-fried foods study ... 3

Table S1. Demographics of the 15 participants in this study ... 5

Table S2. List of 11 MAC standards with their structures ... 6

Table S3. Structures and structural elucidation results of MPhMA, 5HPnFuMA, and 4MSfBAToMMA ... 10

Table S4. List of 847 features mapped to at least one MAC structure in urine samples. ... Separate XLSX file.

Table S5. MACs with significant changes on the fourth day (with structural formulas). ... 11

Table S6. MACs with significant changes on the fifth day (with structural formulas). ... 26

Figure S1. Efficiency of aminoacylase-1 in removing acetyl groups from 11 MAC standards under different amounts of aminoacylase-1. *p < 0.05; **p < 0.01 ... 35

Figure S2. Efficiency of aminoacylase-1 in removing acetyl groups from 11 MAC standards under different incubation times. *p < 0.05; **p < 0.01 ... 37

Figure S3. Comparison of features detected using various isolation window modes and AIF analysis. ... 39

Figure S4. MS/MS spectra of chloramphenicol under different isolation windows. ... 40

Figure S5. Comparison of N-Acetyl-S-(3-carboxy-2-propyl)-L-cysteine between standard and urine samples in chromatograms and MS/MS spectra ... 41

Figure S6. Comparison of N-Acetyl-S-propyl-L-cysteine in the standard and urine samples on the chromatogram and MS/MS spectra. ... 42

Figure S7. Cohen's d values of all annotated MACs with significant abundance change between urine samples collected under deep-fried food consumption and control conditions. ... 43

Figure S8. 95% confidence intervals of each annotated MAC with significant abundance change between urine samples collected under deep-fried food consumption and control conditions on day 4. 44

Figure S9. 95% confidence intervals of each annotated MAC with significant abundance change between urine samples collected under deep-fried food consumption and control conditions on day 5. 45

Figure S10. MAC filtering and structure identification process of deep-fried foods study. 46

Experimental detail of deep-fried foods study

Urine samples were collected from 15 participants on the fourth and fifth day of the first and second weeks of the study. The sampling time was designed to capture both the acute and residual metabolic responses to dietary exposure. Specifically, urine collected on the fourth day was intended to reflect the immediate metabolic output after several consecutive days of exposure to deep-fried foods, during which the levels of short-lived MACs would be the most pronounced. Collection on the fifth day, following the termination of exposure, aimed to detect more persistent MACs or those derived from xenobiotics with longer biological half-lives, offering insight into the body's delayed or sustained metabolic response. This sampling strategy enables a comparative analysis across pre-exposure, active exposure, and post-exposure phases. Both enzymatically treated and untreated samples were prepared and analyzed using HRMS-DIA. A total of 41,300 features were detected across all samples in negative ionization mode. The mass errors of the data were ensured by four nonconjugated internal standards with the average relative mass error = -0.23 ppm. The selected mass tolerance of ± 5 ppm was greater than the standard deviation of the mass error, indicating that the possibility of false negative results could be minimized. MAC candidates were filtered through a combination of neutral loss filtering and signal abundance comparisons between enzymatically treated and untreated samples. The number of features selected at each stage of the filtering process is illustrated in Figure S10.

For neutral loss filtering, 2,411 features exhibited a specific neutral loss in the

deconvoluted MS/MS spectra, with the mass tolerance lower than 5 ppm. In signal abundance analysis, 1,327 features showed a significant decrease after enzymatic treatment (paired Student's t-test, p-value < 0.05, and fold change < 0.5) and were directly classified as MAC candidates. Additionally, 337 features displayed a significant increase (paired Student's t-test, p-value < 0.05, and fold change > 2) and were considered potential signals corresponding to the deacetylated products of MACs. Parent compounds for these deacetylated product candidates (N = 726) were also filtered as MAC candidates. Enzymatic treatment approach filtered overall 2,021 MAC candidates. After combining the enzymatic-treatment and neutral-loss filtering results as a union set, 4,126 MAC candidates were retained and subjected to structural annotation using SIRIUS software. Using the SIRIUS software and the generated MAC structural database, 847 MAC candidate features were mapped to at least one MAC structure with a top-ranking score.

Table S1. Demographics of the 15 participants in this study.

Characteristics	Participants (N = 15)
Gender	
Female	4
Male	11
Age (Mean ± SD years)	24.0 ± 1.6
Height (Mean ± SD m)	172.3 ± 6.1
Weight (Mean ± SD kg)	72.3 ± 18.5
BMI (Mean ± SD kg/m^2)	24.1 ± 5.2

Table S2. List of 11 MAC standards with their structures.

Compound name	SMILES	Structure
N-Acetyl-S-phenyl-L-cysteine (SPMA)	CC(=O)N[C@@H](CSC1=CC=CC=C1)C(=O)O	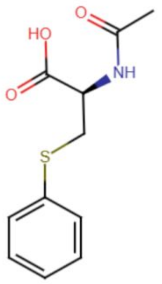
N-Acetyl-S-benzyl-L-cysteine (BzMA)	CC(=O)N[C@@H](CSCC1=CC=CC=C1)C(=O)O	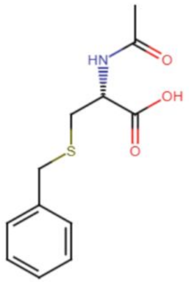

N-Acetyl-S-(1,2-dichloroethenyl)-L-cysteine (12CEMA)	CC(=O)N[C@@H](CS/C(=C/Cl)/Cl)C(=O)O	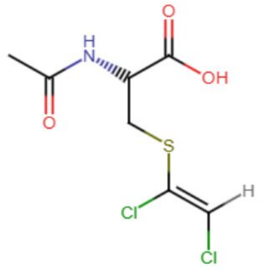
N-Acetyl-S-(2,4-dimethylphenyl)-L-cysteine (MPhMA)	CC1=CC(=C(C=C1)SC[C@@H](C(=O)O)NC(=O)C)C	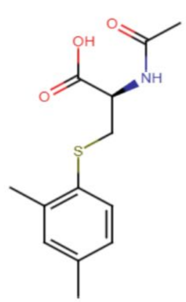
N-Acetyl-S-(4-nitrophenyl)-L-cysteine (4NPhMA)	CC(=O)N[C@@H](CSC1=CC=C(C=C1)[N+](=O)[O-])C(=O)O	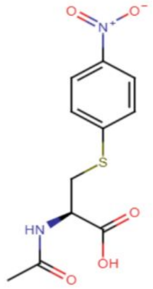

N-Acetyl-S-(trichlorovinyl)-L-cysteine (122CVMA)	CC(=O)N[C@@H](CSC(=C(Cl)Cl)Cl)C(=O)O	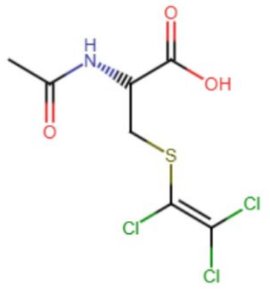
N-Acetyl-S-(tetrahydro-5-hydroxy-2- pentyl-3-furanyl)-L-cysteine (5HPnFuMA)	CCCCCC1C(CC(O1)O)SC[C@@H](C(=O)O)NC(=O)C	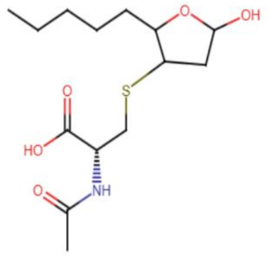
N-Acetyl-S-[N-(2-phenylethyl)thiocarbamoyl]-L-cysteine (2PhECaMA)	CC(=O)N[C@@H](CSC(=S)NCCC1=CC=CC=C1)C(=O)O	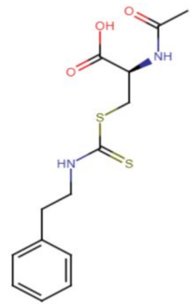

N-Acetyl-S-(2,4-dinitrophenyl)-L-cysteine (24NPhMA)	CC(=O)N[C@@H](CSC1=C(C=C(C=C1)[N+](=O)[O-])[N+](=O)[O-])C(=O)O	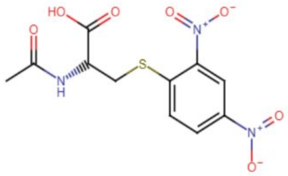
N-Acetyl-S-[[[4-(methylsulfinyl)butyl]amino]thioxomethyl]-L-cysteine (4MSfBAToMMA)	CC(=O)N[C@H](CSC(=S)NCCCCS(=O)C)C(=O)O	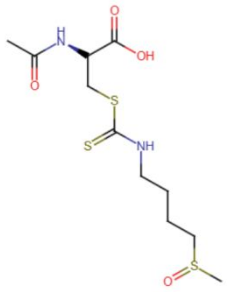
N-Acetyl-S-trans,trans-farnesyl-L-cysteine (AFC)	CC(=CCC/C(=C/CC/C(=C/CSC[C@@H](C(=O)O)NC(=O)C)/C)/C)C	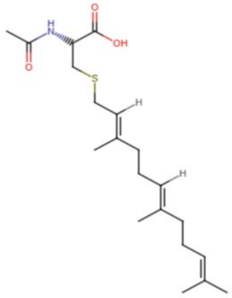

Table S3. Structures and structural elucidation results of MPhMA, 5HPnFuMA, and 4MSfBAToMMA. In method validation, 3 of the 11 MAC standards did not match their top-ranked candidate from structural elucidation. However, these incorrect structures shared similarity to their actual structures.

	MPhMA	5HPnFuMA	4MSfBAToMMA
The structures of MAC standards	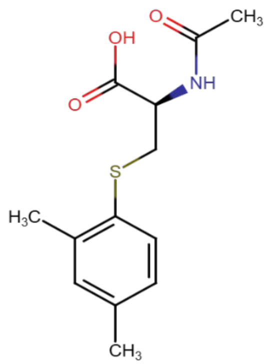	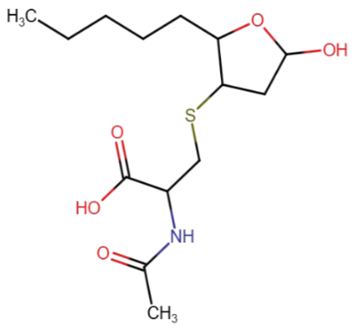	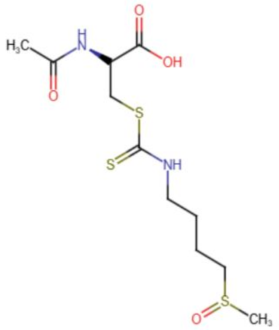
The structural elucidation results	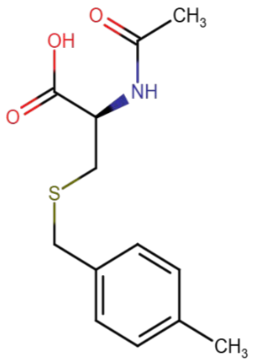	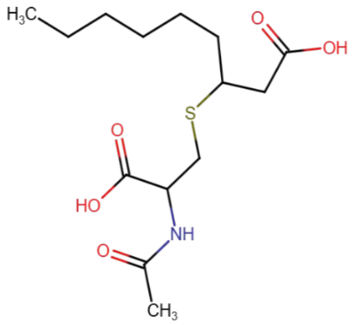	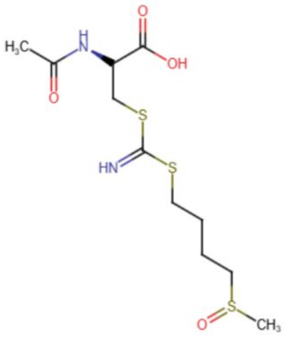

Table S5. MACs with significant changes on the fourth day (with structural formulas).

m/z	RT (min)	Structure of MACs	Molecular formula of MACs	Fold change (log_2)	Paired t-test *p*-value
422.1853	6.5	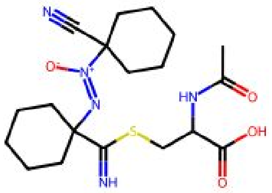	$C_{19}H_{29}N_5O_4S$	3.6	0.020
318.0654	3.3	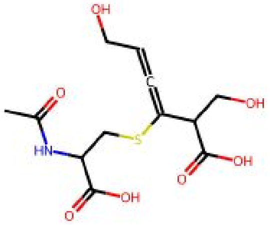	$C_{12}H_{17}NO_7S$	2.9	0.027
461.1670	4.5	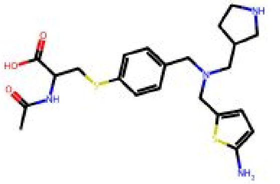	$C_{22}H_{30}N_4O_3S_2$	2.7	0.010

m/z	RT (min)	Structure of MACs	Molecular formula of MACs	Fold change (log_2)	Paired t-test *p*-value
429.1199	6.3	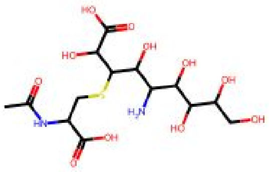	$C_{14}H_{26}N_2O_{11}S$	2.6	0.044
311.0417	1.0	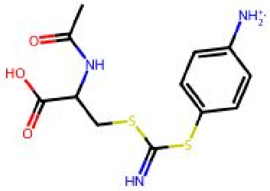	$C_{12}H_{14}N_3O_3S_2$	2.3	0.006
335.1620	6.2	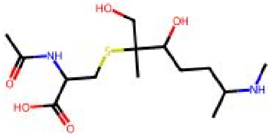	$C_{14}H_{28}N_2O_5S$	2.3	0.004

m/z	RT (min)	Structure of MACs	Molecular formula of MACs	Fold change (log_2)	Paired t-test *p*-value
254.0863	1.9	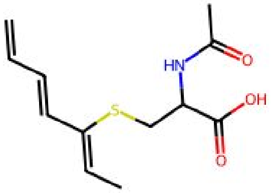	$C_{12}H_{17}NO_3S$	2.1	0.013
461.1665	4.6	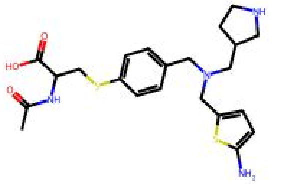	$C_{22}H_{30}N_4O_3S_2$	2.1	0.026
350.1627	7.1	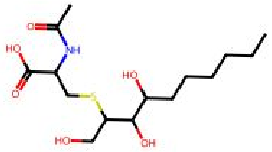	$C_{15}H_{29}NO_6S$	2.1	0.014

m/z	RT (min)	Structure of MACs	Molecular formula of MACs	Fold change ($\log_2$)	Paired t-test *p*-value
402.1956	7.4	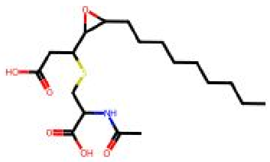	$C_{19}H_{33}NO_6S$	2.0	0.002
366.1010	6.0	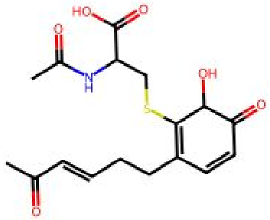	$C_{17}H_{21}NO_6S$	1.9	0.002
332.1539	6.2	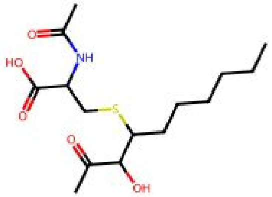	$C_{15}H_{27}NO_5S$	1.9	0.001

m/z	RT (min)	Structure of MACs	Molecular formula of MACs	Fold change (log_2)	Paired t-test *p*-value
358.0596	4.9	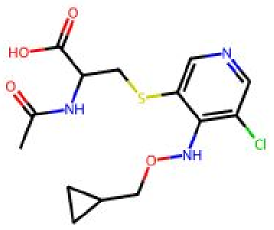	$C_{14}H_{18}ClN_3O_4S$	1.8	0.021
380.1356	5.6	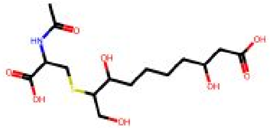	$C_{15}H_{27}NO_8S$	1.7	0.016
290.1434	5.4	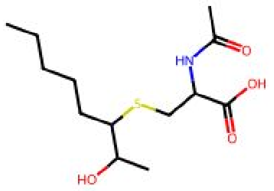	$C_{13}H_{25}NO_4S$	1.7	0.021

m/z	RT (min)	Structure of MACs	Molecular formula of MACs	Fold change (log_2)	Paired t-test *p*-value
330.1386	6.4	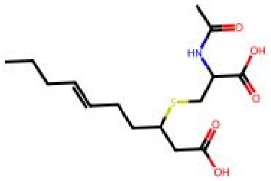	$C_{15}H_{25}NO_5S$	1.7	0.022
364.1435	5.2	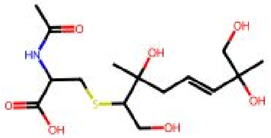	$C_{15}H_{27}NO_7S$	1.7	0.046
362.1648	6.6	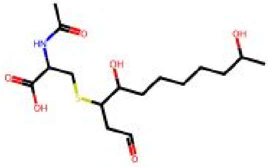	$C_{16}H_{29}NO_6S$	1.7	0.007

m/z	RT (min)	Structure of MACs	Molecular formula of MACs	Fold change ($\log_2$)	Paired t-test *p*-value
374.1437	6.2	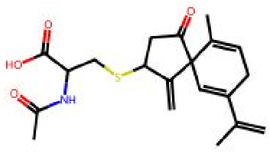	$C_{20}H_{25}NO_4S$	1.7	0.038
367.0784	8.2	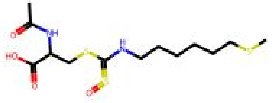	$C_{13}H_{24}N_2O_4S_3$	1.7	0.008
290.1435	5.5	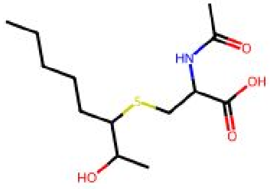	$C_{13}H_{25}NO_4S$	1.6	0.006

m/z	RT (min)	Structure of MACs	Molecular formula of MACs	Fold change (log_2)	Paired t-test *p*-value
332.1539	6.1	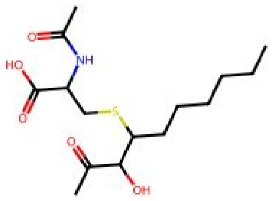	$C_{15}H_{27}NO_5S$	1.6	0.001
377.1574	7.6	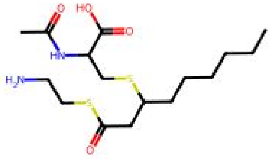	$C_{16}H_{30}N_2O_4S_2$	1.6	0.019
343.9696	5.9	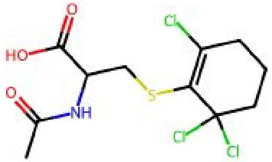	$C_{11}H_{14}Cl_3NO_3S$	1.5	0.009

m/z	RT (min)	Structure of MACs	Molecular formula of MACs	Fold change (log_2)	Paired t-test *p*-value
593.1918	4.9	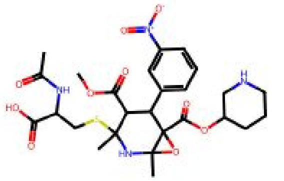	$C_{26}H_{34}N_4O_{10}S$	1.5	0.034
234.0439	2.0	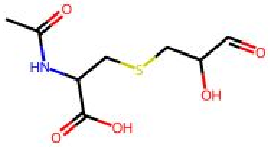	$C_8H_{13}NO_5S$	1.4	0.030
307.1127	5.9	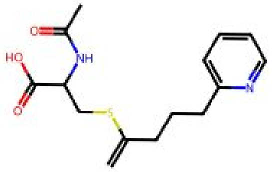	$C_{15}H_{20}N_2O_3S$	1.4	0.017

m/z	RT (min)	Structure of MACs	Molecular formula of MACs	Fold change (log_2)	Paired t-test *p*-value
234.0444	2.1	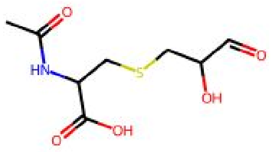	$C_8H_{13}NO_5S$	1.4	0.036
333.1568	5.5	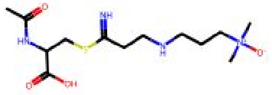	$C_{13}H_{26}N_4O_4S$	1.4	0.012
259.0755	5.2	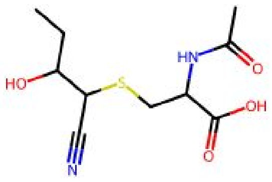	$C_{10}H_{16}N_2O_4S$	1.4	0.012

m/z	RT (min)	Structure of MACs	Molecular formula of MACs	Fold change (log_2)	Paired t-test *p*-value
290.1433	7.0	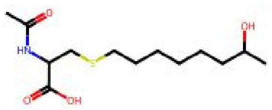	$C_{13}H_{25}NO_4S$	1.3	0.024
356.1355	7.5	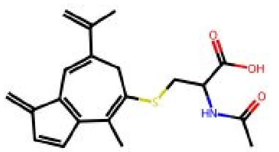	$C_{20}H_{23}NO_3S$	1.3	0.005
315.0849	5.9	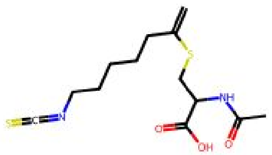	$C_{13}H_{20}N_2O_3S_2$	1.3	0.011

m/z	RT (min)	Structure of MACs	Molecular formula of MACs	Fold change (log2)	Paired t-test *p*-value
387.0931	4.7	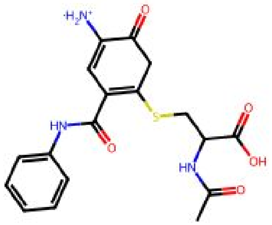	$C_{18}H_{18}N_3O_5S$	1.3	0.043
306.1018	4.2	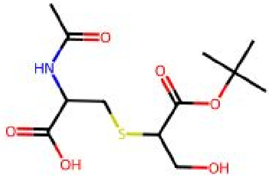	$C_{12}H_{21}NO_6S$	1.2	0.040
328.1224	6.9	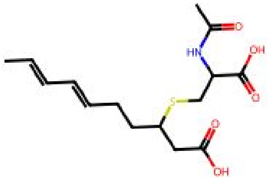	$C_{15}H_{23}NO_5S$	1.2	0.037

m/z	RT (min)	Structure of MACs	Molecular formula of MACs	Fold change (log_2)	Paired t-test *p*-value
364.1441	5.0	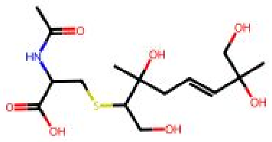	$C_{15}H_{27}NO_7S$	1.2	0.016
318.1384	6.0	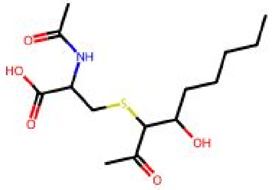	$C_{14}H_{25}NO_5S$	1.2	0.024
243.0442	2.7	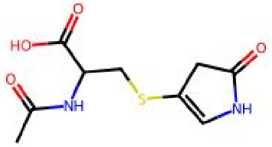	$C_9H_{12}N_2O_4S$	1.1	0.018

m/z	RT (min)	Structure of MACs	Molecular formula of MACs	Fold change (log_2)	Paired t-test *p*-value
379.1027	6.8	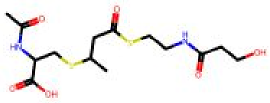	$C_{14}H_{24}N_2O_6S_2$	1.1	0.044
304.1593	7.9	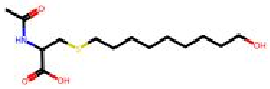	$C_{14}H_{27}NO_4S$	1.0	0.000
325.0947	0.9	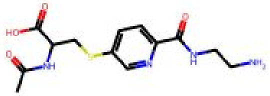	$C_{13}H_{18}N_4O_4S$	-1.1	0.004

m/z	RT (min)	Structure of MACs	Molecular formula of MACs	Fold change (log2)	Paired t-test *p*-value
354.0931	0.9	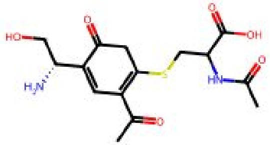	$C_{15}H_{19}N_2O_6S$	-1.3	0.032
290.0690	1.0	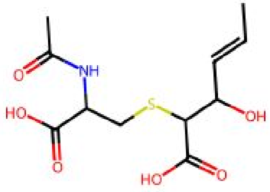	$C_{11}H_{17}NO_6S$	-1.5	0.009
242.0105	0.9	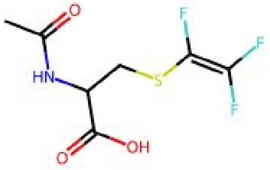	$C_7H_8F_3NO_3S$	-1.6	0.017

Table S6. MACs with significant changes on the fifth day (with structural formulas).

m/z	RT (min)	Structure of MACs	Molecular formula of MACs	Fold change (log_2)	Paired t-test *p*-value
363.1928	7.0	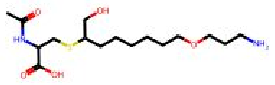	$C_{16}H_{32}N_2O_5S$	4.4	0.014
332.1268	5.0	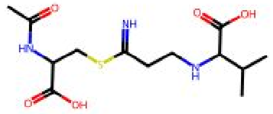	$C_{13}H_{20}N_3O_5S$	2.4	0.017
445.0787	6.2	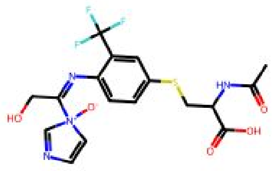	$C_{17}H_{17}F_3N_4O_5S$	1.9	0.022

m/z	RT (min)	Structure of MACs	Molecular formula of MACs	Fold change (log_2)	Paired t-test *p*-value
382.0969	6.2	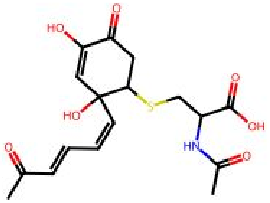	$C_{17}H_{21}NO_7S$	1.9	0.003
372.1117	5.7	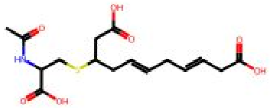	$C_{16}H_{23}NO_7S$	1.7	0.008
513.0652	6.1	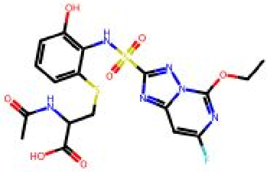	$C_{18}H_{19}FN_6O_7S_2$	1.6	0.030

m/z	RT (min)	Structure of MACs	Molecular formula of MACs	Fold change (log_2)	Paired t-test *p*-value
296.0963	6.1	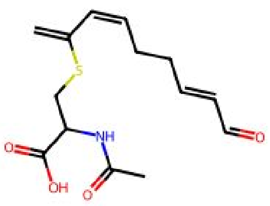	$C_{14}H_{19}NO_4S$	1.5	0.013
260.0326	3.3	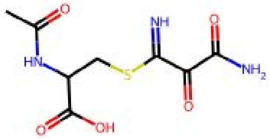	$C_8H_{11}N_3O_5S$	1.4	0.021
442.1538	6.0	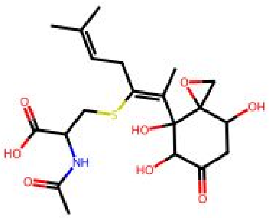	$C_{20}H_{29}NO_8S$	1.3	0.014

m/z	RT (min)	Structure of MACs	Molecular formula of MACs	Fold change (log_2)	Paired t-test *p*-value
295.0667	1.0	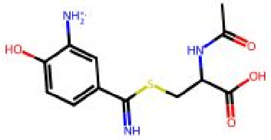	$C_{12}H_{14}N_3O_4S$	1.3	0.006
442.1539	5.8	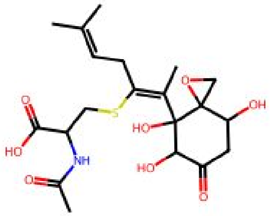	$C_{20}H_{29}NO_8S$	1.2	0.002
494.2032	6.5	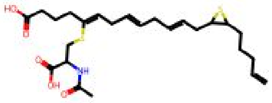	$C_{25}H_{37}NO_5S_2$	1.2	0.010

m/z	RT (min)	Structure of MACs	Molecular formula of MACs	Fold change (log_2)	Paired t-test *p*-value
388.1606	5.1	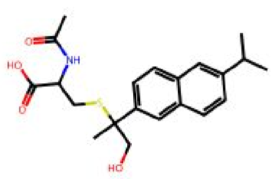	$C_{21}H_{27}NO_4S$	1.1	0.036
383.1895	7.7	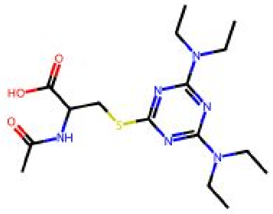	$C_{16}H_{28}N_6O_3S$	1.1	0.038
358.0608	5.1	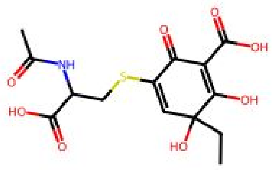	$C_{14}H_{17}NO_8S$	1.1	0.021

m/z	RT (min)	Structure of MACs	Molecular formula of MACs	Fold change (log_2)	Paired t-test *p*-value
325.0947	0.9	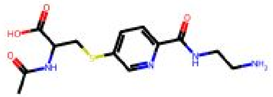	$C_{13}H_{18}N_4O_4S$	1.1	0.044
350.1627	7.1	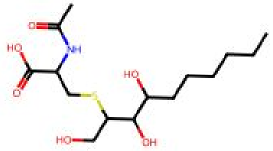	$C_{15}H_{29}NO_6S$	1.0	0.040
377.0546	3.8	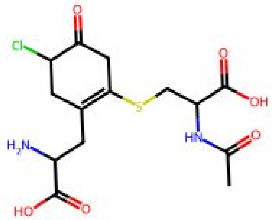	$C_{14}H_{19}ClN_2O_6S$	1.0	0.023

m/z	RT (min)	Structure of MACs	Molecular formula of MACs	Fold change (log_2)	Paired t-test *p*-value
295.0450	1.1	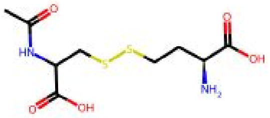	$C_9H_{16}N_2O_5S_2$	-1.0	0.012
349.0645	1.1	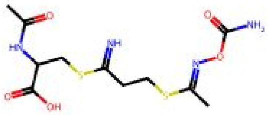	$C_{11}H_{18}N_4O_5S_2$	-1.1	0.006
290.0690	1.0	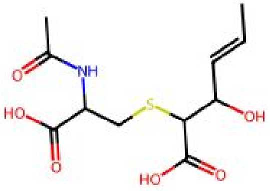	$C_{11}H_{17}NO_6S$	-1.1	0.042

m/z	RT (min)	Structure of MACs	Molecular formula of MACs	Fold change (log_2)	Paired t-test *p*-value
309.0909	6.7	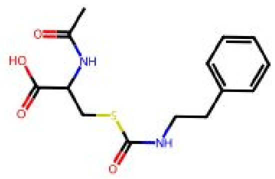	$C_{14}H_{18}N_2O_4S$	-1.1	0.009
340.0574	6.6	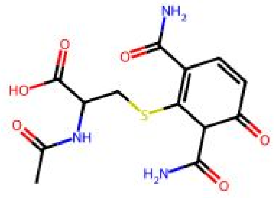	$C_{13}H_{15}N_3O_6S$	-1.1	0.013
295.0447	1.4	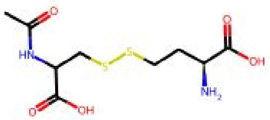	$C_9H_{16}N_2O_5S_2$	-1.2	0.014

m/z	RT (min)	Structure of MACs	Molecular formula of MACs	Fold change (log_2)	Paired t-test *p*-value
190.0176	1.1	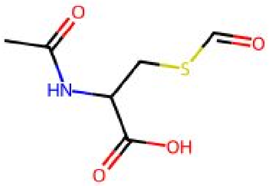	$C_6H_9NO_4S$	-2.2	0.031

Figure S1. Efficiency of aminoacylase-1 in removing acetyl groups from 11 MAC standards under different amounts of aminoacylase-1. *p < 0.05; **p < 0.01

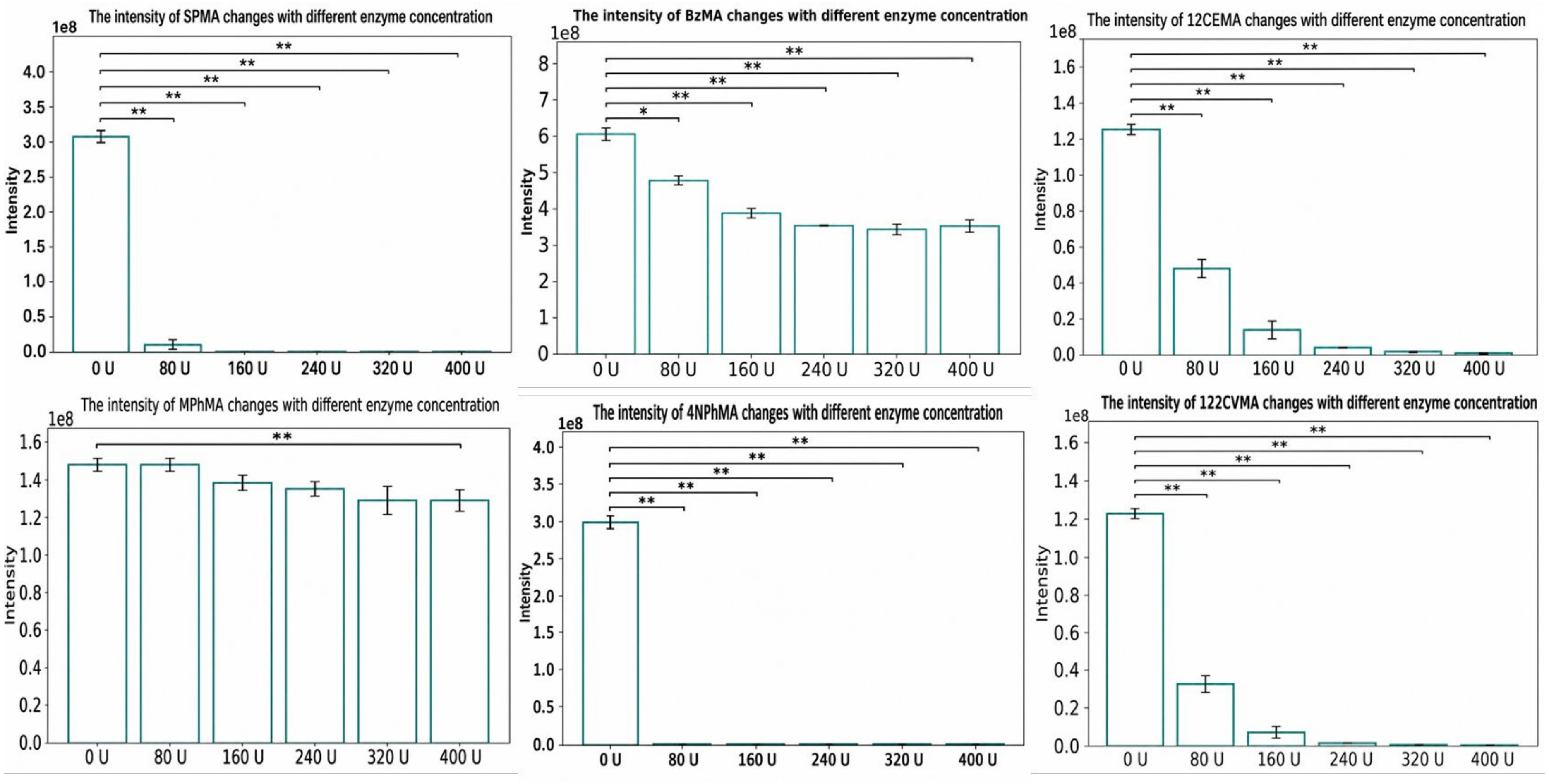

Figure S1 continue

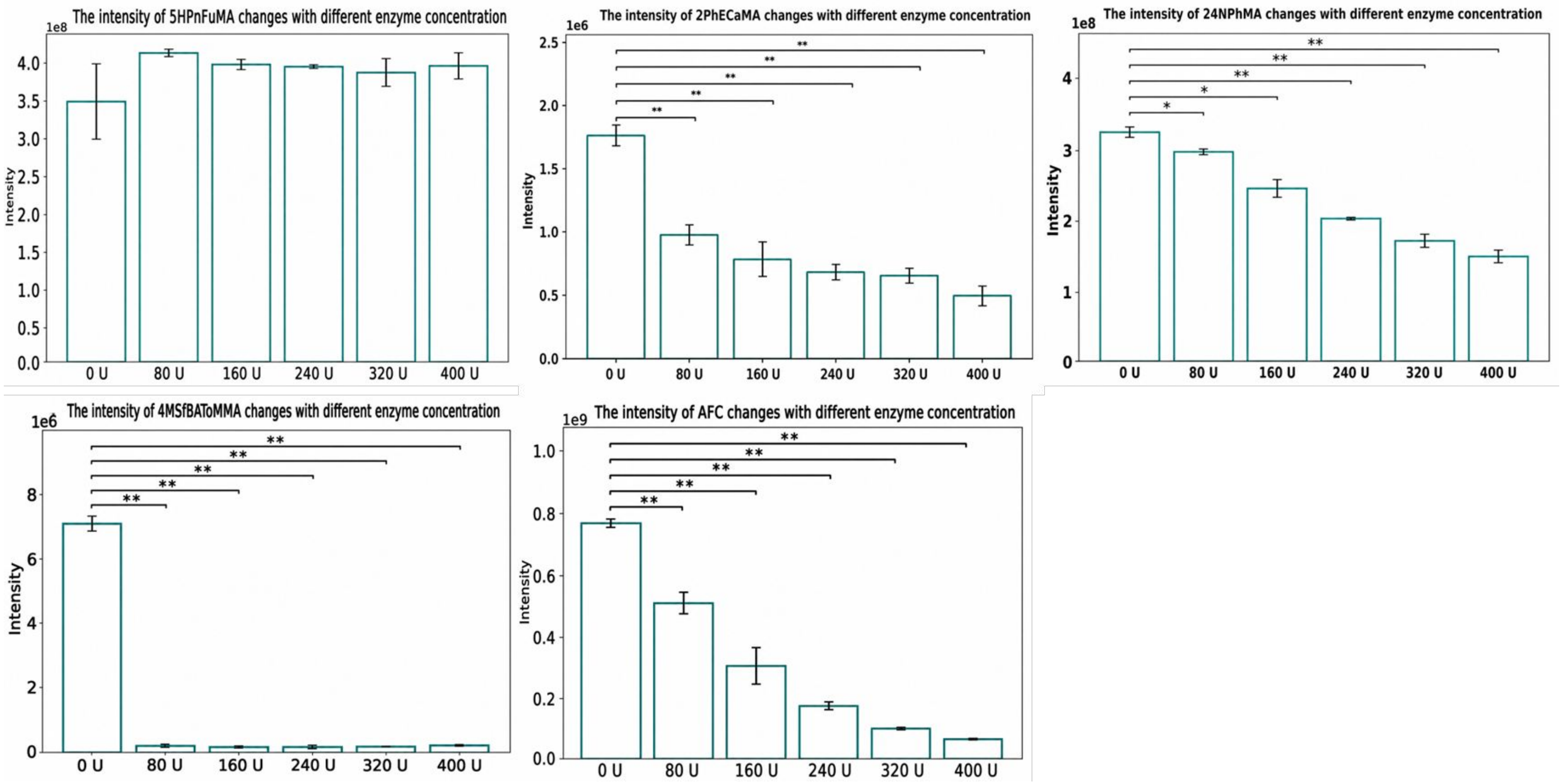

Figure S2. Efficiency of aminoacylase-1 in removing acetyl groups from 11 MAC standards under different incubation times. *p < 0.05; **p < 0.01

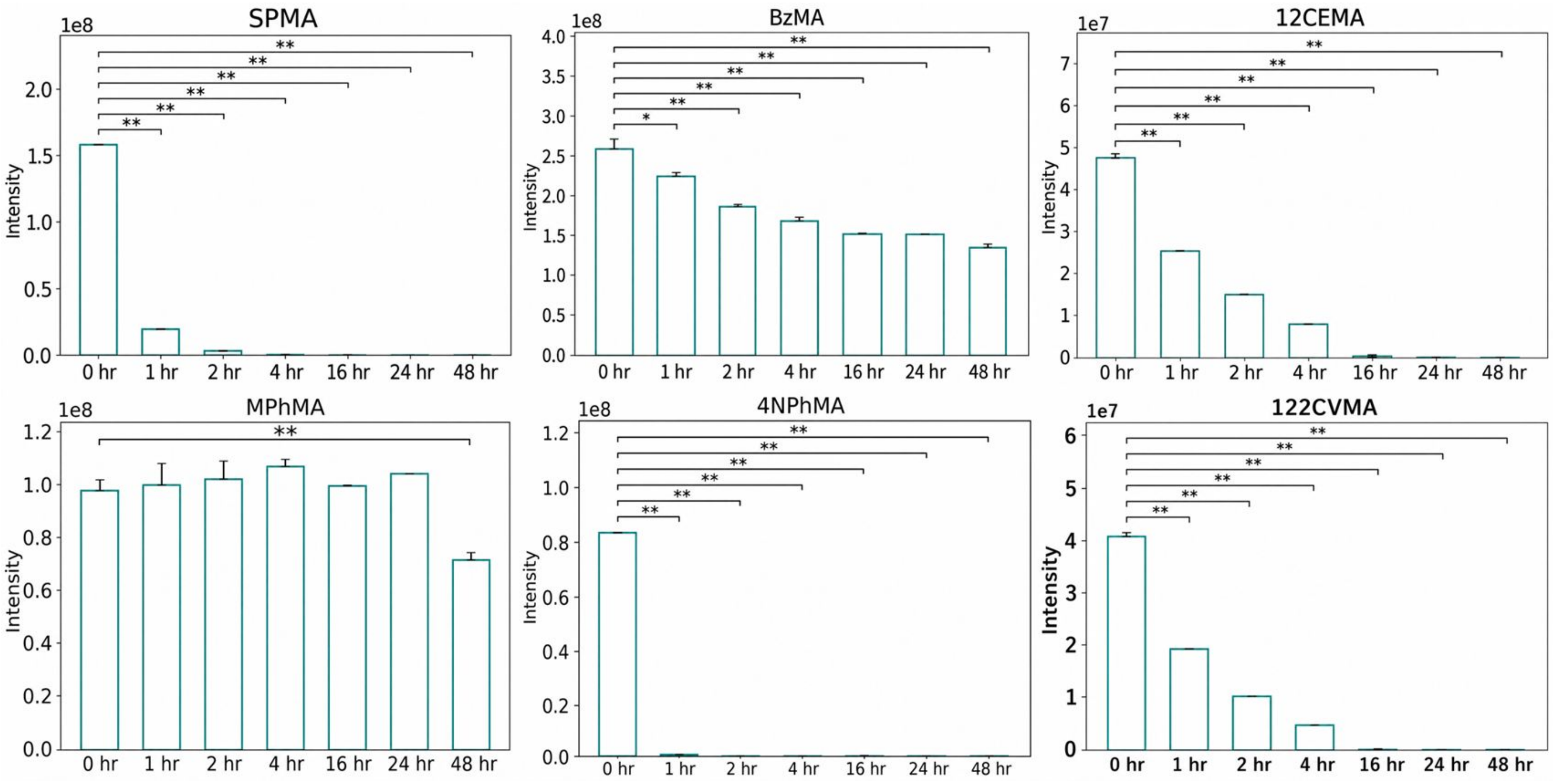

Figure S2 continue

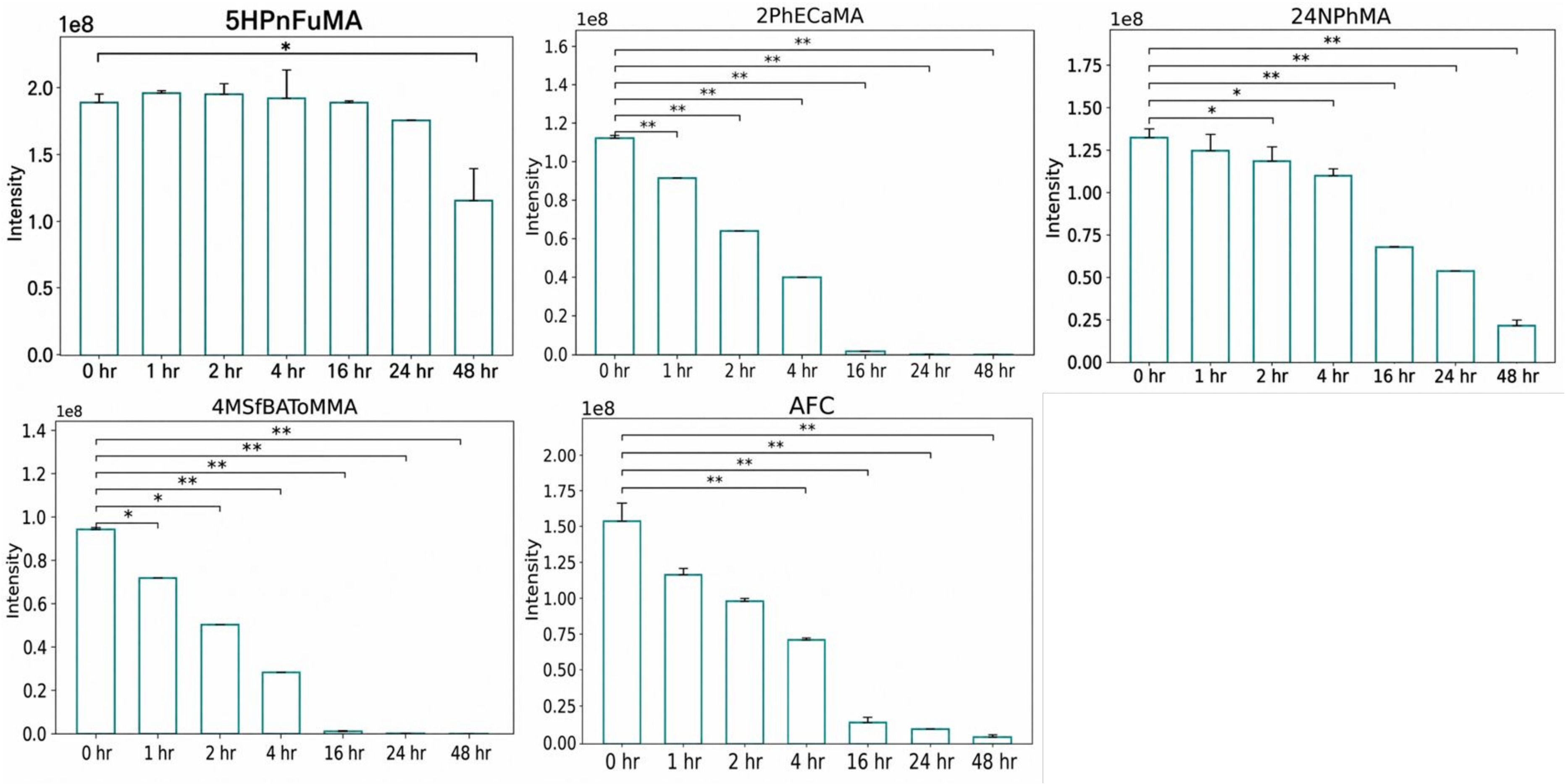

Figure S3. Comparison of features detected using various isolation window modes and AIF analysis. Urine samples were analyzed under different DIA acquisition modes with isolation window widths of 60, 80, and 120 *m/z*, as well as AIF analysis. AIF analysis yielded the highest feature count (21,106 features), followed closely by DIA mode with a 120 *m/z* window (20,664 features).

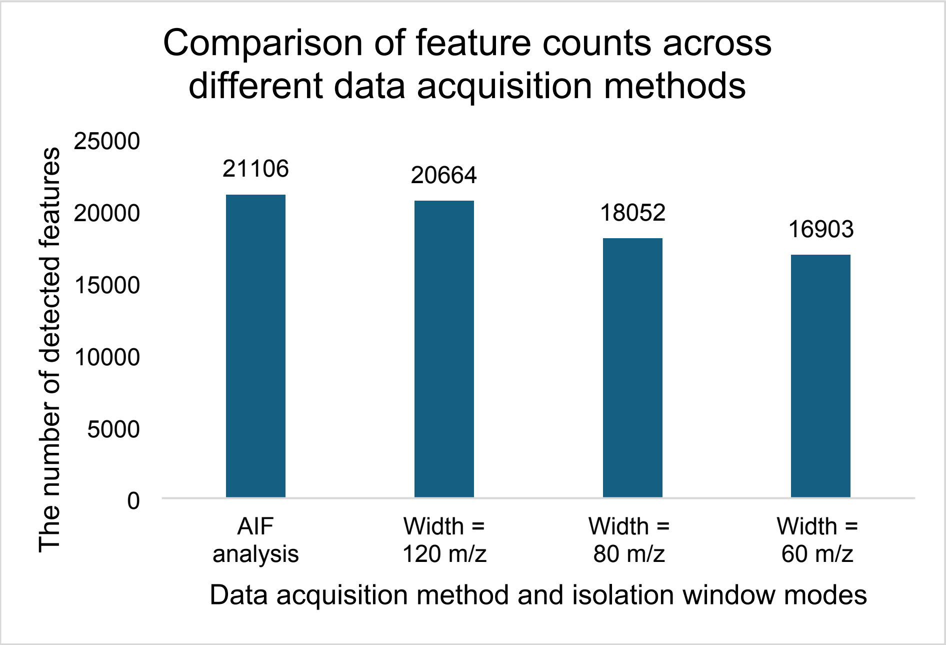

Figure S4. MS/MS spectra of chloramphenicol under different isolation windows. The MS/MS spectra of chloramphenicol, one of the internal standards, were analyzed to evaluate the quality of spectra obtained with isolation window widths of 60, 80, and 120 *m/z* in DIA mode, as well as in AIF mode. The red dotted line represents the actual fragments observed in the DDA MS/MS spectrum. MS/MS spectra obtained with DIA isolation window widths of 60, 80, and 120 *m/z* exhibit comparable quality. The AIF spectrum exhibited substantial baseline noise.

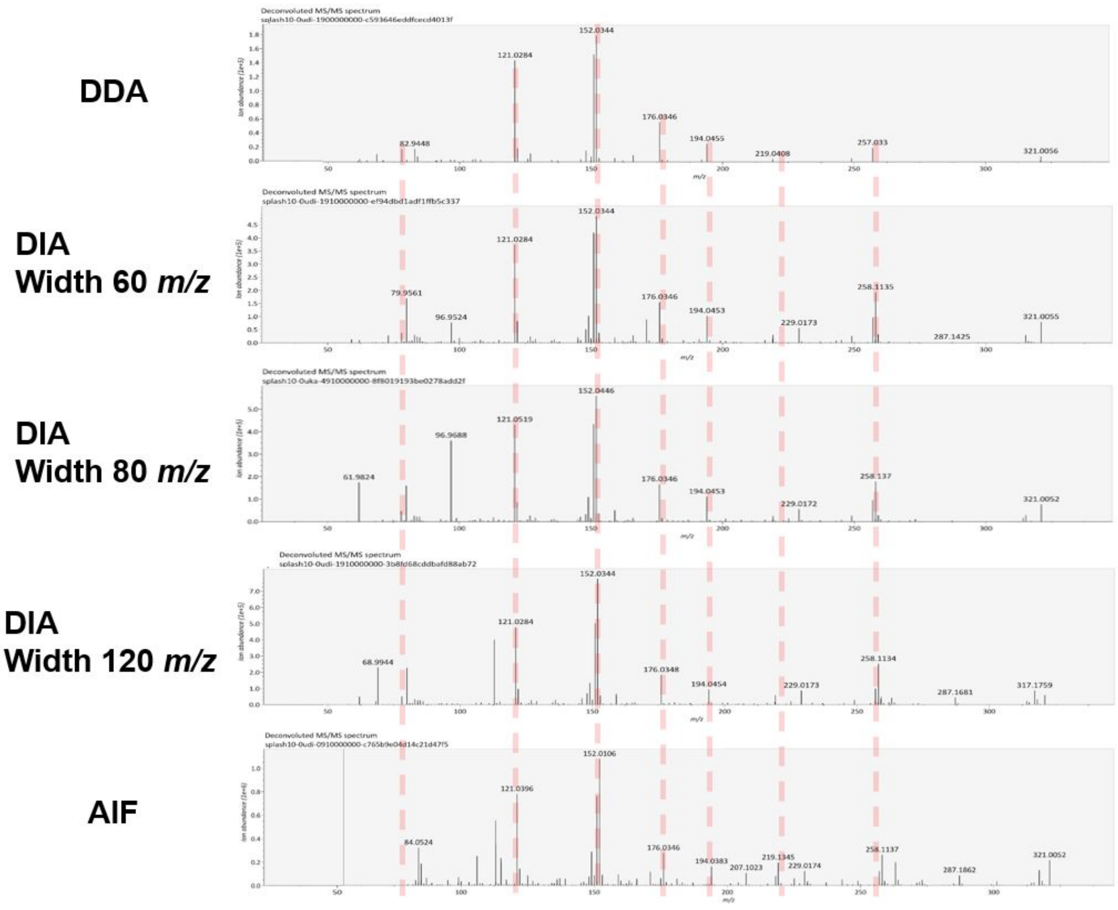

Figure S5. (A) Comparison of N-Acetyl-S-(3-carboxy-2-propyl)-L-cysteine between standard and urine samples in chromatograms. The chemical structure displayed alongside the spectra of urine sample represents the top-ranked in silico structural candidate predicted by the SIRIUS software. (B) Comparison of N-Acetyl-S-(3-carboxy-2-propyl)-L-cysteine in standard and urine samples on MS/MS spectra.

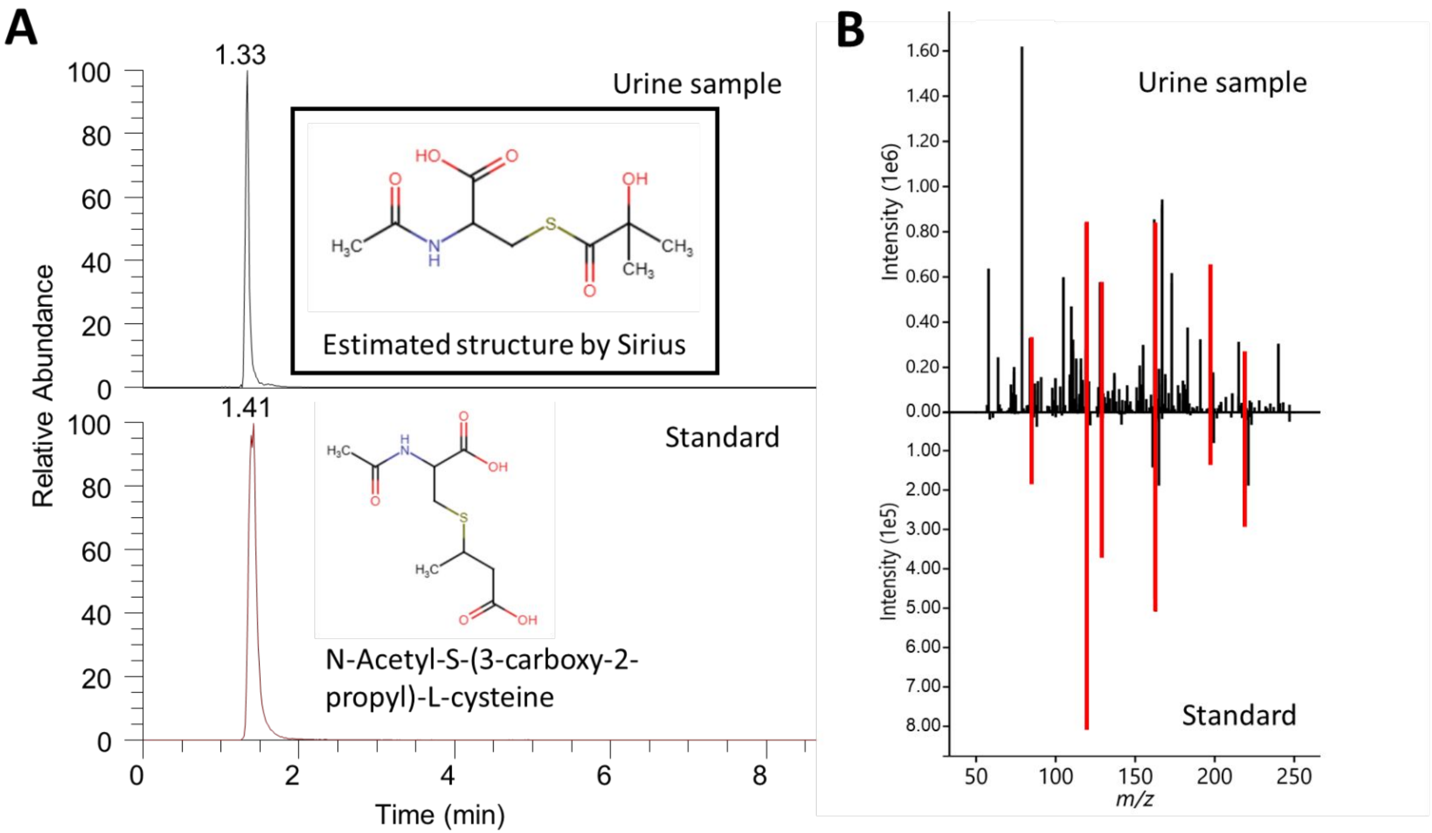

Figure S6. (A) Comparison of N-Acetyl-S-propyl-L-cysteine in the standard and urine samples on the chromatogram. The chemical structure displayed alongside the spectra of urine sample represents the top-ranked in silico structural candidate predicted by the SIRIUS software. (B) Comparison of N-Acetyl-S-propyl-L-cysteine in standard and urine samples on MS/MS spectra.

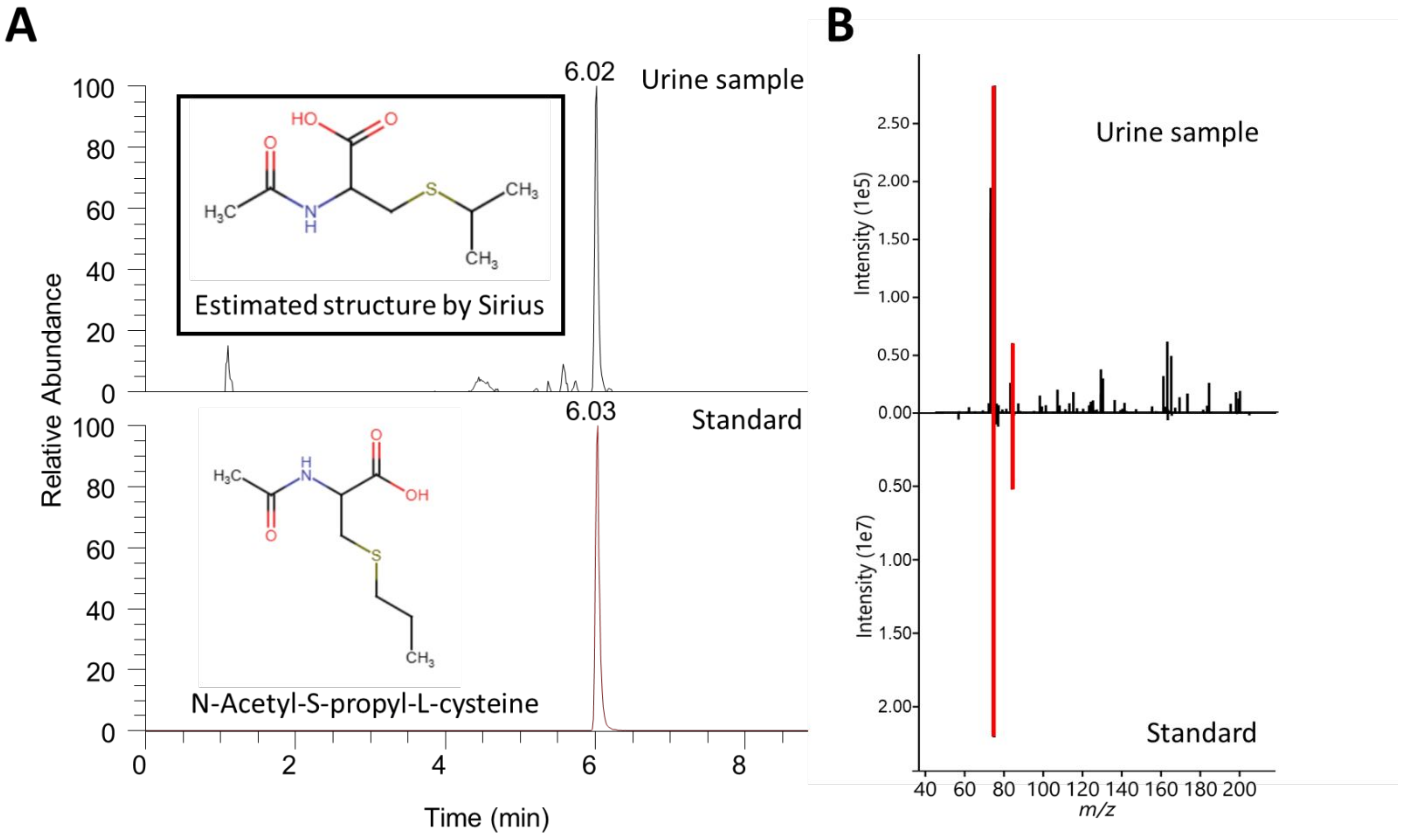

Figure S7. Cohen's d values of all annotated MACs with significant abundance change between urine samples collected under deep-fried food consumption and control conditions.

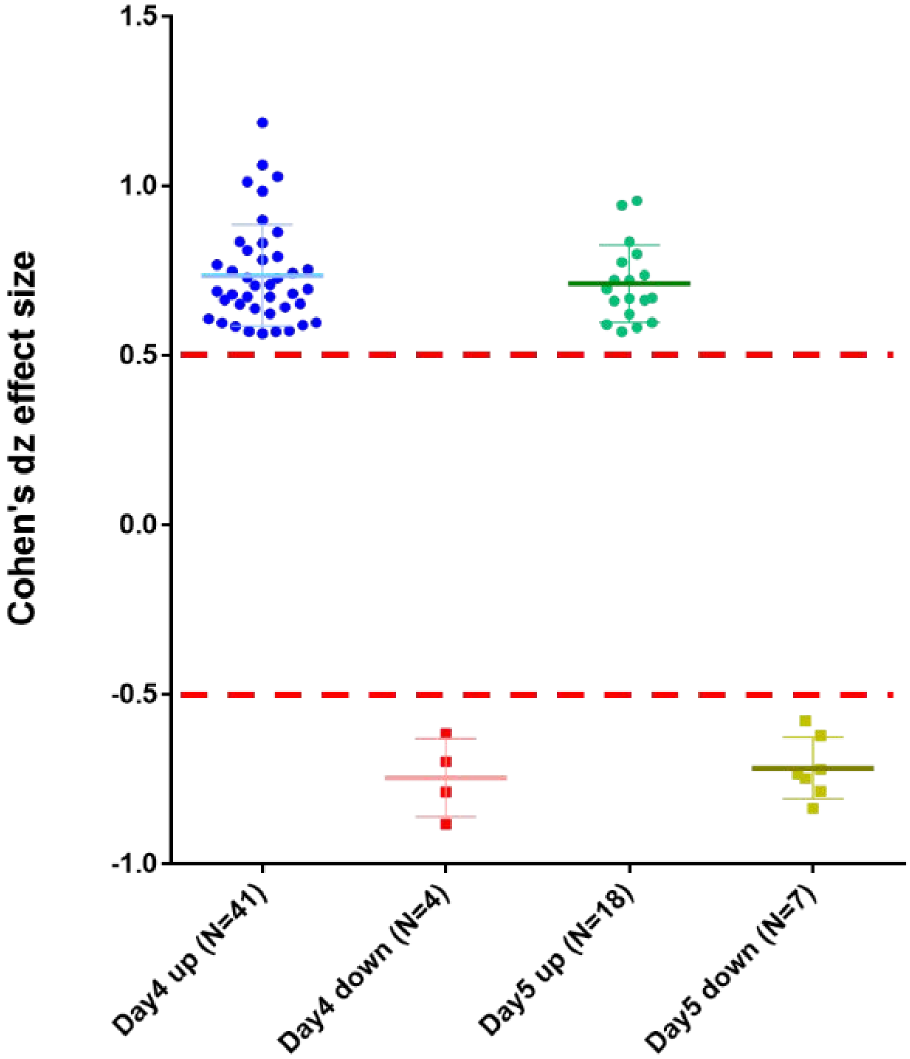

Figure S8. 95% confidence intervals of each annotated MAC with significant abundance change between urine samples collected under deep-fried food consumption and control conditions on day 4.

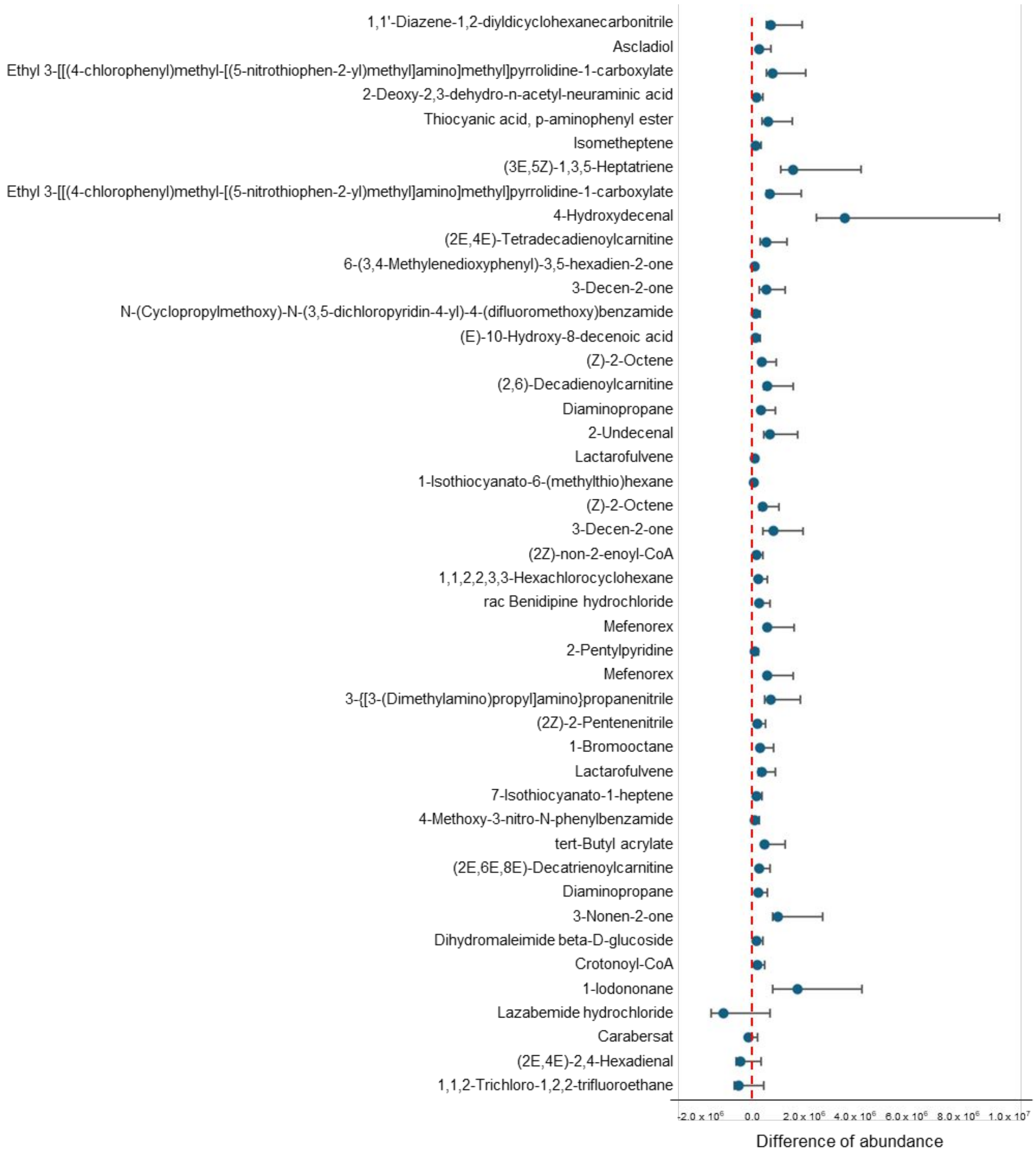

Figure S9. 95% confidence intervals of each annotated MAC with significant abundance change between urine samples collected under deep-fried food consumption and control conditions on day 5.

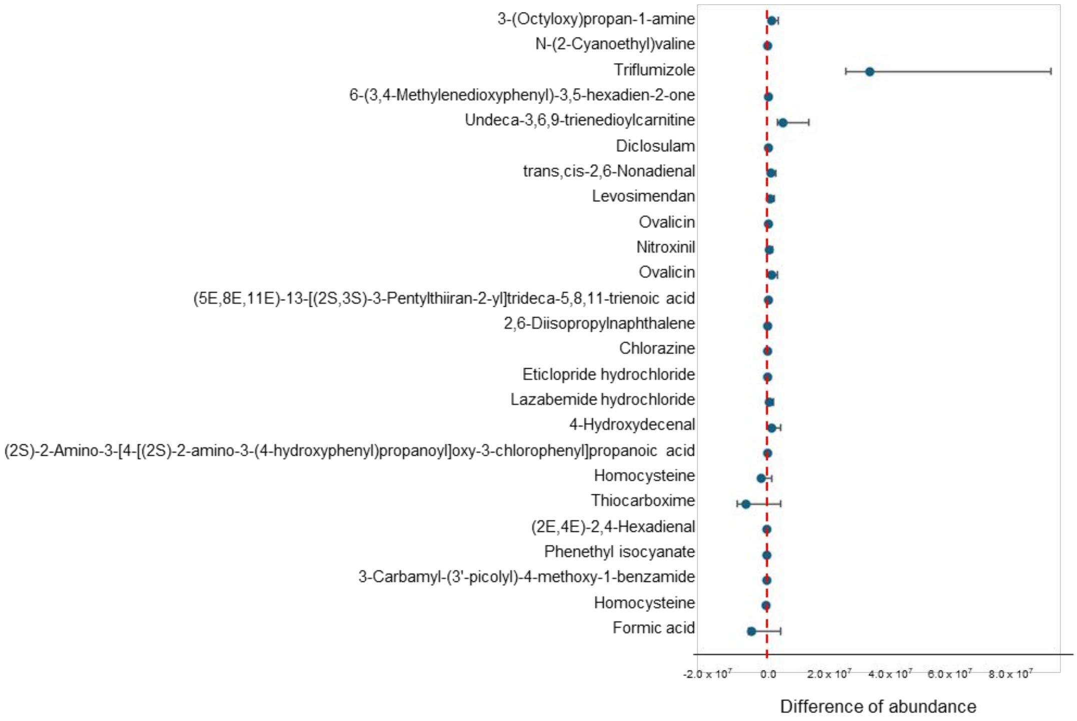

Figure S10. MAC filtering and structure identification process of deep-fried foods study

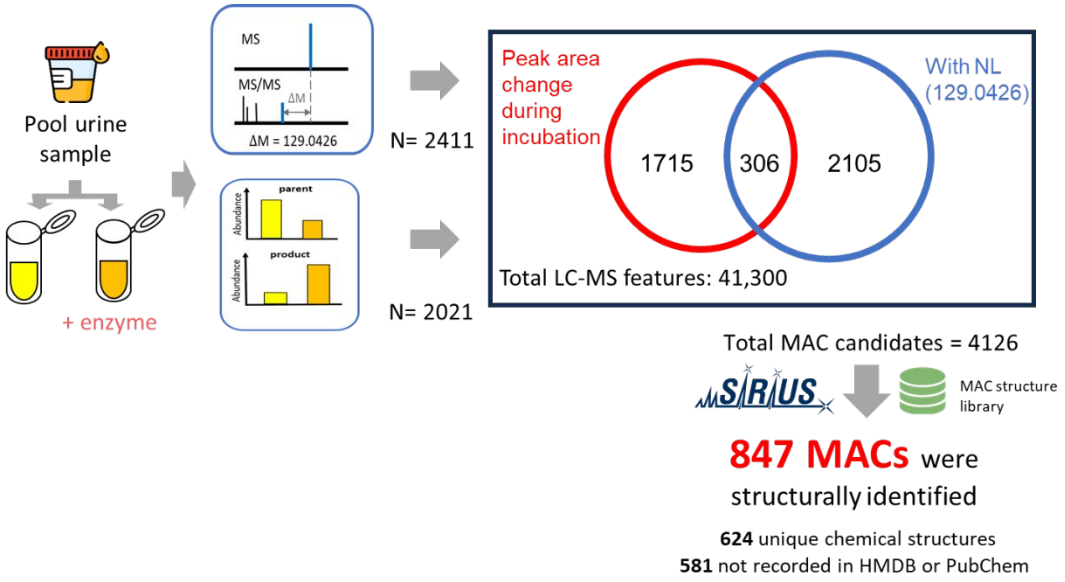

Supplement: Supplementary file 1 [file ac5c08181_si_001.pdf]
